# Supplementary material for: Brain neuromarkers predict self- and other-related mentalizing across adult, clinical, and developmental samples
Source: Nat Commun. 2026 Jun 6;17:7229. doi: 10.1038/s41467-026-73945-w (PMC13396484; doi:10.1038/s41467-026-73945-w)
Supplement: Supplementary file 1 — Supplementary Information [file 41467_2026_73945_MOESM1_ESM.pdf]

Supplementary Materials

Brain neuromarkers predict self- and other-related mentalizing across adult, clinical, and developmental samples

Dorukhan Açıl, Jessica Andrews-Hanna, Marina Lopez-Sola, Mariët van Buuren, Lydia Krabbendam, Liwen Zhang, Lisette van der Meer, Paola Fuentes-Claramonte, Edith Pomarol-Clotet, Raymond Salvador, Martin Debbané, Pascal Vrticka, Patrik Vuilleumier, David A. Sbarra, Andrea M. Coppola, Anita Tusche, Lars O. White, Tor D. Wager, & Leonie Koban*

*Please address correspondence to:

Dr. Leonie Koban, CRNL, Institut des Épilepsies IDEE, 59 Boulevard Pinel, 69500 Bron, France; email: leonie.koban@cnrs.fr

Content: 2 supplementary figures; 7 supplementary tables.

Supplementary Table 1

Demographics

Study	Population	n	Gender (females)	Age mean (SD)	Handedness (left)	Education (in years)	Place	References
Study 1	Healthy adults	21	10	23.7 (7.4)	2	N/A	Switzerland	Koban et al., 2014
Study 2	Adolescents	44	23	16.0 (1.9)	0	N/A	Switzerland	Debbane et al., 2017
Study 3	Adolescents	61	27	12.9 (0.4)	11	N/A	The Netherlands	van Buuren et al., 2020
Study 4a	Healthy adults	33	14	41.7 (11.7)	0	12.7	Spain	Fuentes-Claramonte et al., 2019 & 2020
Study 4b	Schizophrenia patients	23	7	37.0 (8.1)	0	10.4		
Study 5a	Healthy adults	15	6	33.3 (11.3)	2	17.2	The Netherlands	Zhang et al., 2015
Study 5b	Schizophrenia patients	17	6	35.5 (9.7)	1	16.6		
Study 5c	Bipolar patients	18	10	40.3 (12.7)	0	17.0		
Study 6a	Healthy adults	59	26	28.3 (5.2)	2	N/A	USA	Tusche et al., 2023
Study 6b	Healthy adults	50	19	33.6 (7.3)	4	N/A		
Study 7	Healthy adults	49	26	22.7 (3.9)	6	13	USA	Ma et al., 2024
**Overall**		**390**	**174**	**26.9 (11.7)**	**28**			

Note. N/A = not assessed or inaccessible. Links to the published studies are as follows: Study 2 (doi.org/10.3758/s13415-017-0497-9); Study 3 (doi.org/10.1016/j.neuroimage.2020.117060); Study 4, 2019 (doi.org/10.1371/journal.pone.0209376); Study 4, 2020 (doi.org/10.1016/j.nicl.2019.102134); Study 5 (doi.org/10.1016/j.nicl.2015.04.010); Study 6 (doi.org/10.1038/s41467-023-40078-3); Study 7 (doi.org/10.31234/osf.io/qcj45)

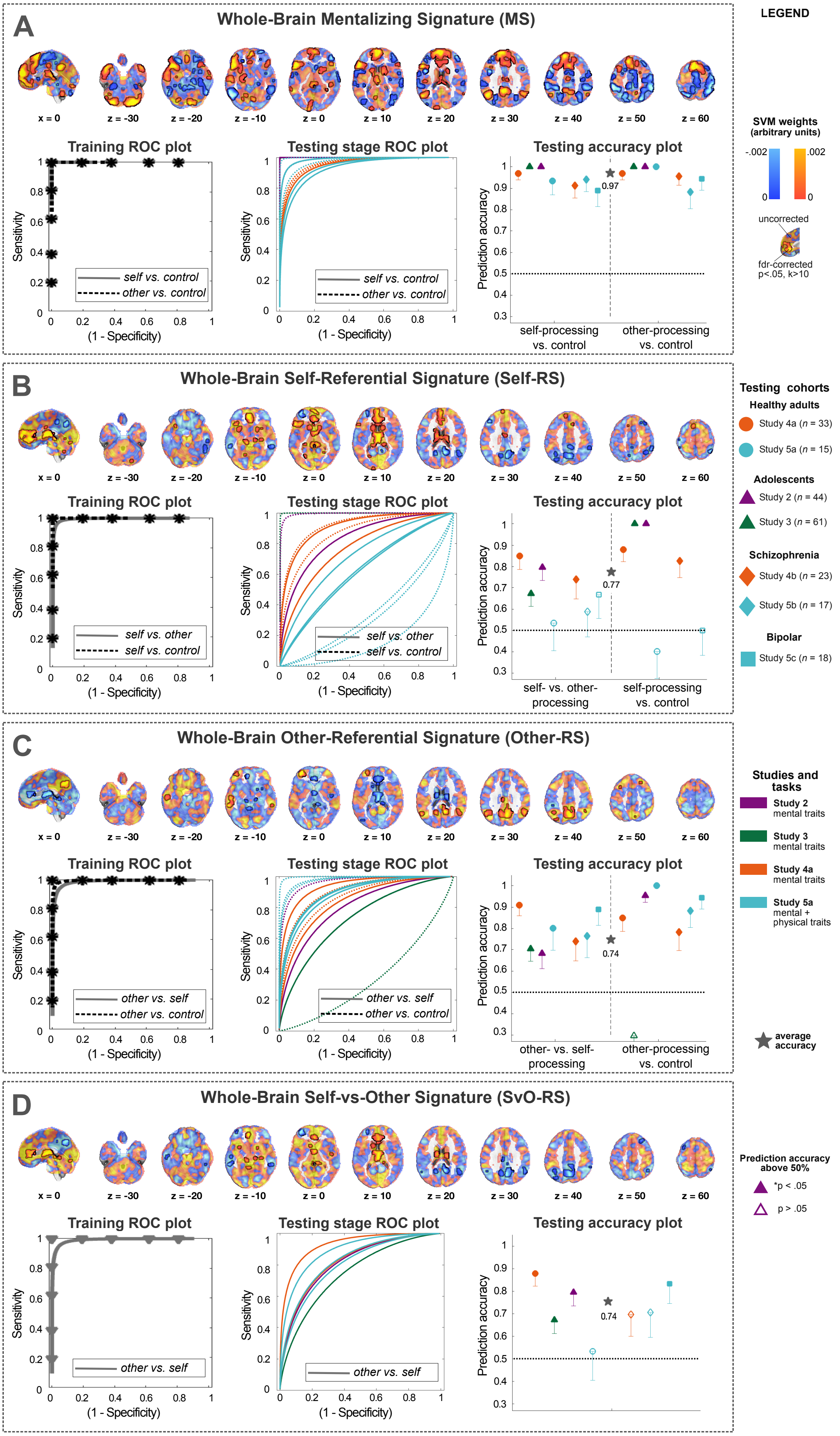
A
Whole-Brain Mentalizing Signature (MS)
B
Whole-Brain Self-Referential Signature (Self-RS)
C
Whole-Brain Other-Referential Signature (Other-RS)
D
Whole-Brain Self-vs-Other Signature (SvO-RS)
x = 0
z = -30
z = -20
z = -10
z = 0
z = 10
z = 20
z = 30
z = 40
z = 50
z = 60
Training ROC plot
Testing stage ROC plot
Testing accuracy plot
Sensitivity
(1 - Specificity)
Prediction accuracy
self vs. control
other vs. control
self vs. other
other vs. self
0.97
0.77
0.74
self-processing vs. control
other-processing vs. control
self- vs. other-processing
other- vs. self-processing
LEGEND
SVM weights (arbitrary units)
-.002
.002
0
uncorrected
fdr-corrected p<.05, k>10
Testing cohorts
Healthy adults
Study 4a (n = 33)
Study 5a (n = 15)
Adolescents
Study 2 (n = 44)
Study 3 (n = 61)
Schizophrenia
Study 4b (n = 23)
Study 5b (n = 17)
Bipolar
Study 5c (n = 18)
Studies and tasks
Study 2 mental traits
Study 3 mental traits
Study 4a mental traits
Study 5a mental + physical traits
average accuracy
Prediction accuracy above 50%
*p < .05
p > .05

Supplementary Figure 1 — Whole brain classifiers (*n* = 21). In order to determine whether the use of a social-cognition mask may have altered the results substantially, we trained new classifiers using the same analytic flow (*n* = 21) but using whole-brain contrast images (with a grey-matter mask). We trained SVM classifiers using 5000 bootstrap samples derived from *n* = 21 subjects, yielding statistical maps with two-tailed, uncorrected p-values for A) the whole-brain Mentalizing Signature, B) the whole-brain Self-Referential Signature, C) the whole-brain Other-Referential Signature, and D) the whole-brain Self-vs-Other Signature. On the top row of each box, weight maps illustrate the uncorrected positive and negative weights for each classifier. Voxels significant at an FDR-corrected threshold ($q < .05$, minimum cluster size of $k = 10$) are highlighted by black outlines. The left and middle plots in each box depict the receiver operating characteristic (ROC) plots from the i) cross-validated training and ii) testing datasets. The last column shows the two-alternative forced-choice prediction accuracies of all three mentalizing signatures in each testing dataset separately (total *n* = 211), with color representing the study, and shape illustrating the cohort type. Filled shapes indicate classification accuracies significantly above chance level (50%) according to two-sided binomial tests at $p < .05$; unfilled shapes indicate non-significant accuracies. The one-sided error bars below the shapes illustrate the standard error around the accuracy estimates. The star shows the weighted average accuracy across all seven datasets. These additional analyses illustrate that masking the contrast images used in this study did not alter the results substantially, as the whole-brain approach resulted in comparable prediction accuracies and brain weights.

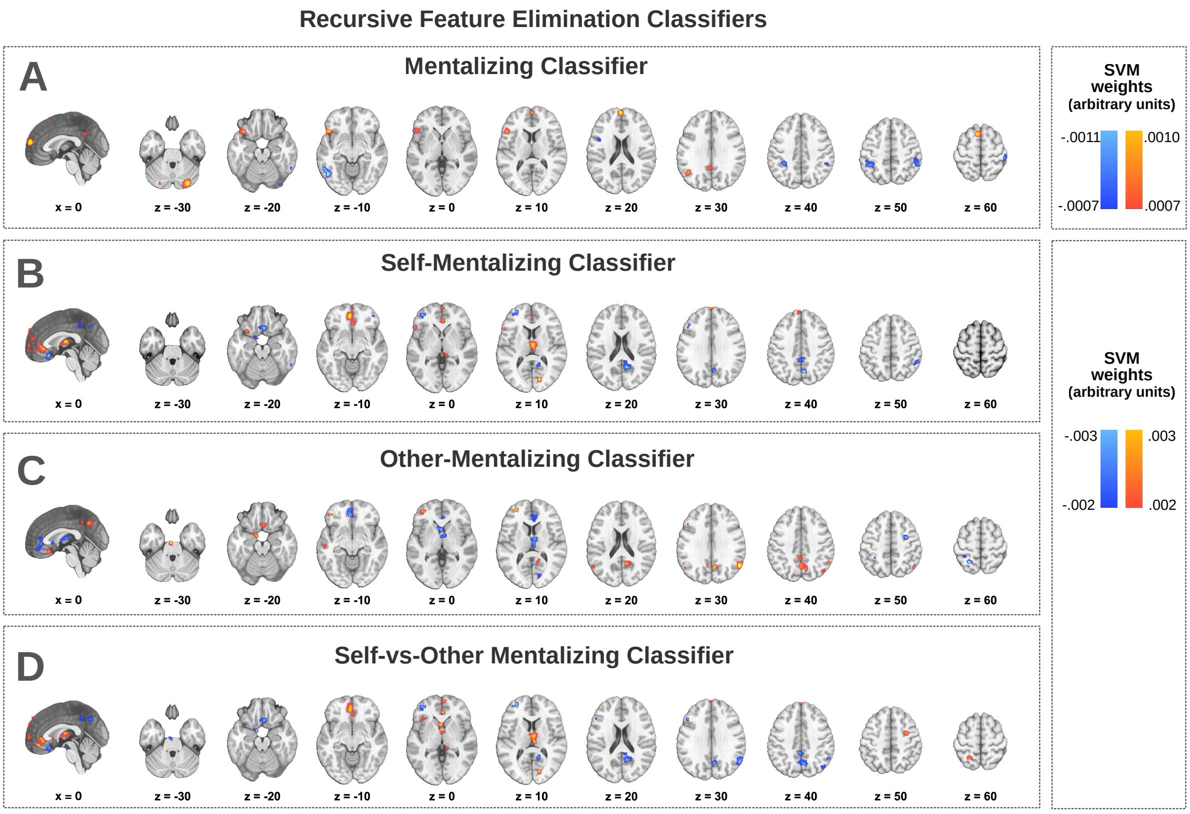

Supplementary Figure 2 — Recursive feature elimination (RFE) classifiers (*n* = 21). Additional classifiers using a recursive feature elimination (RFE) method yielded results similar to those obtained with the original bootstrapping approach. Specifically, we developed four classifiers using RFE, for A) the overall Mentalizing classifier, B) the Self-Mentalizing classifier, C) the Other-Mentalizing classifier, and D) the Self-versus-Other Mentalizing classifier. Linear SVM models with 10-fold cross-validation were trained in multiple iterations, in which 5000 voxels were removed from the model in each step to reach the final voxel size of 2000. The final models with 2000 features are illustrated here, including only clusters with a minimum voxel size of 20. All four final models had 100% (two-alternative forced-choice) prediction accuracies in the cross-validated training phase (binomial tests at $p < .05$ threshold).

Supplementary Table 2

Significant clusters of the Self-RS

Labeled name	Atlas region (see note)	x	y	z	Voxels	Max(z)
Cerebellum	Cblm Crus II R	26	-80	-38	54	0.00035
Ventral anterior cingulate cortex (vACC) / ventromedial prefrontal cortex (vmPFC) (incl. dorsomedial and frontopolar prefrontal cortices)	Multiple regions	-2	44	0	933	0.00068
Anterior insula (AI)	AAIC L	-34	10	-10	33	0.00043
Caudate nucleus	Caudate Ca R	18	10	-6	13	0.00033
Inferior frontal gyrus (IFG)	45 L	-46	24	2	162	0.00057
Thalamus	Multiple regions	0	-12	10	311	0.00068
Caudate nucleus	Caudate Ca R	12	14	8	17	0.00031
Caudate nucleus	Caudate Ca L	-14	14	6	32	0.00035
Dorsolateral prefrontal cortex (dlPFC)	9m L	-8	48	38	21	0.00037
Superior frontal lobule / dorsomedial prefrontal cortex (dmPFC)	SFL L	-8	22	56	46	0.00040
Temporal pole	TGd L	-34	14	-34	13	-0.00050
Inferior temporal gyrus/sulcus	TE2p R	46	-46	-22	27	-0.00045
Posterior temporal cortex	PH R	52	-54	-14	21	-0.00054
dlPFC	a9 46v L	-38	48	4	26	-0.00072
Posterior cingulate cortex (PCC)	POS2 R	6	-60	32	270	-0.00070
Inferior parietal cortex (IPC) / temporoparietal junction (TPJ)	PGi R	52	-62	22	52	-0.00046
IPC / TPJ	PFm R	52	-50	24	20	-0.00035
IPC / TPJ	PGs L	-34	-76	40	14	-0.00044
PCC	31a L	-2	-42	42	20	-0.00054
IPC / TPJ	PFm R	52	-44	48	28	-0.00064

Note. Significant positive and negative weights contributing to the Self-Referential Signature (Self-RS). SVM classifiers were trained using 5000 bootstrap samples which produced two-tailed, uncorrected *p*-values. We applied an FDR-correction ($q < 0.05$ with cluster size $k > 10$) across the masked whole-brain to illustrate regions that contributed to the models most strongly. L refers to the left hemisphere, and R refers to the right hemisphere. Cortical atlas regions are labeled based on a combination of parcellations available on GitHub: https://github.com/canlab/Neuroimaging_Pattern_Masks/tree/master/Atlases_and_parcellations/2018_Wager_combined_atlas.

Supplementary Table 3

Significant clusters of the Other-RS

Labeled name	Atlas region (see note)	x	y	z	Voxels	Max(z)
Superior temporal sulcus (STS)	STSda L	-56	-16	-12	174	0.00053
dlPFC	a9 46v L	-38	50	2	90	0.00085
PCC / Precuneus	Multiple regions	4	-60	34	733	0.00081
IPC / TPJ	PGi L	-52	-60	26	245	0.00048
IPC / TPJ	PGi R	52	-58	30	339	0.00058
Retrosplenial cortex	RSC L	-4	-28	28	15	0.00039
IPC / TPJ	PGs L	-36	-76	42	14	0.00048
IPC / TPJ	PFm L	-42	-58	42	38	0.00034
Anterior insula (AI)	AAIC R	30	12	-10	15	-0.00034
vACC / vmPFC	Multiple regions	-2	40	2	498	-0.00058
Caudate nucleus	Caudate Ca R	20	8	-6	20	-0.00032
Thalamus	Bstem Midbd R	12	-30	-2	24	-0.00049
Anterior insula (AI)	AAIC L	-34	10	-6	13	-0.00027
Ventral striatum	V Striatum L	-2	16	-2	13	-0.00045
Thalamus	Multiple regions	0	-10	10	314	-0.00059
Caudate nucleus	Caudate Ca L	-12	14	6	24	-0.00036
Ventral striatum	Cau R	0	24	8	13	-0.00035
Middle temporal complex (MT+)	LO3 L	-44	-80	12	17	-0.00026
IPC	IP2 L	-46	-40	42	10	-0.00036
Somatosensory cortex	2 L	-30	-44	54	19	-0.00033

Note. Significant positive and negative weights contributing to the Other-Referential Signature (MS-Other). SVM classifiers were trained using 5000 bootstrap samples which produced two-tailed, uncorrected *p*-values. We applied an FDR-correction ($q < 0.05$ with cluster size $k > 10$) across the masked whole-brain to illustrate regions that contributed to the models most strongly. L refers to the left hemisphere, and R refers to the right hemisphere. Cortical atlas regions are labeled based on a combination of parcellations available on GitHub: https://github.com/canlab/Neuroimaging_Pattern_Masks/tree/master/Atlases_and_parcellations/2018_Wager_combined_atlas.

Supplementary Table 4

Significant clusters of the MS

Labeled name	Atlas region (see note)	x	y	z	Voxels	Max(z)
Cerebellum	Cblm IX R	2	-54	-44	109	0.00016
Cerebellum	Cblm CrusI R	28	-82	-32	597	0.00040
Cerebellum	Cblm CrusI L	-24	-80	-34	373	0.00028
STS/IFG (incl. vlPFC, AI, STG, temporal pole)	Multiple regions	-48	18	-10	2536	0.00038
Temporal pole	TGd R	50	16	-30	114	0.00014
Orbitofrontal cortex (OFC)	10v R	0	38	-20	15	0.00012
IFG / ventrolateral PFC (vlPFC)	45 R	44	24	-10	195	0.00014
Amygdala	Bstem Ponscd	-24	-12	-10	68	0.00010
Dorsal anterior cingulate cortex (dACC) / vACC (incl. dmPFC, vmPFC, dlPFC, frontal eye fields)	Multiple regions	-6	42	36	3532	0.00036
STS	STSdp R	46	-28	0	20	0.00010
Putamen	Putamen Pa R	30	12	0	13	0.00007
Thalamus	Bstem_SC R	4	-28	2	10	0.00015
Caudate nucleus	Caudate Ca R	16	10	10	107	0.00012
Thalamus	Thal MD	-2	-16	10	96	0.00025
IPC / TPJ	PGi L	-48	-62	26	512	0.00023
Posterior opercular cortex	OP1 L	-50	-24	18	12	0.00008
IPC / TPJ	PGi R	52	-58	30	48	0.00012
PCC	Multiple regions	-2	-50	32	544	0.00023
dlPFC	9p R	18	40	36	42	0.00010
Supplementary motor area (SMA)	8Av L	-36	12	46	68	0.00012
dlPFC	8BL R	14	26	52	19	0.00008
Perirhinal entorhinal cortex	PeEc L	-34	0	-38	17	-0.00010
Amygdala	Amygdala LB	26	0	-26	13	-0.00012
Cerebellum	Cblm VI L	-36	-42	-24	21	-0.00011
Middle temporal gyrus (MTG) posterior	TE1p R	54	-52	-14	215	-0.00024
MTG anterior	TE2a R	48	-18	-22	27	-0.00012
Hippocampus	H R	20	-16	-20	29	-0.00020
Entorhinal cortex/amygdala	Pir R	36	2	-18	29	-0.00014
Parahippocampal gyrus	PHA3 L	-32	-32	-20	10	-0.00010
Amygdala	Amygdala LB	18	4	-18	29	-0.00015
Posterior temporal cortex / MT+	PH L	-52	-58	-10	419	-0.00029
OFC	11l R	20	54	-14	34	-0.00019
MTG medial	TE1m R	60	-26	-16	12	-0.00011

Posterior temporal cortex	PH R	50	-70	-8	43	-0.00017
Auditory association cortex	A5 R	60	-6	-2	54	-0.00020
STS	STSvp R	56	-40	-4	12	-0.00012
Middle Insula	MI L	-34	14	0	18	-0.00010
Inferior frontal sulcus (IFS)	IFSa R	48	38	6	57	-0.00016
Extrastriate cortex / MTG	TPOJ2 R	48	-64	12	327	-0.00013
Premotor cortex / inferior frontal sulcus	6r L	-46	6	26	333	-0.00023
dlPFC	a9 46v L	-36	48	10	17	-0.00016
Extrastriate cortex	V3CD R	36	-84	12	32	-0.00011
IPC	PGp L	-44	-80	16	38	-0.00009
Superior temporal gyrus (STG)	STV R	58	-42	18	35	-0.00012
Supramarginal gyrus / IPC	PF L	-44	-42	44	629	-0.00029
TPJ / Angular gyrus (AG)	PSL R	60	-30	18	18	-0.00011
dlPFC	p9 46v L	-40	30	22	10	-0.00010
Premotor cortex	6v R	50	14	28	32	-0.00015
IPC	IP1 R	30	-64	42	151	-0.00016
IPC / TPJ	PFm R	46	-42	48	576	-0.00033
PCC	5mv L	-10	-38	46	121	-0.00019
Superior parietal cortex / medial intraparietal sulcus	MIP L	-24	-62	50	75	-0.00018
Middle cingulate cortex	24dd R	10	-16	42	14	-0.00006
SMA	SCEF R	0	4	52	284	-0.00018
Premotor cortex / SMA	55b R	50	-4	44	31	-0.00013
SMA	6a L	-28	0	50	20	-0.00010
SMA	8Av R	32	30	50	13	-0.00021
SMA	6a L	-24	8	54	23	-0.00012
Primary sensory cortex	1 R	46	-28	62	35	-0.00036

Note. Significant positive and negative weights contributing to the Mentalizing Signature (MS). SVM classifiers were trained using 5000 bootstrap samples which produced two-tailed, uncorrected *p*-values. We applied an FDR-correction ($q < 0.05$ with cluster size $k > 10$) across the masked whole-brain to illustrate regions that contributed to the models most strongly. L refers to the left hemisphere, and R refers to the right hemisphere. Cortical atlas regions are labeled based on a combination of parcellations available on GitHub: https://github.com/canlab/Neuroimaging_Pattern_Masks/tree/master/Atlases_and_parcellations/2018_Wager_combined_atlas.

Supplementary Table 5

Significant clusters of the Self-vs-Other Referential Signature (SvO-RS)

Labeled name	Atlas region (see note)	x	y	z	Voxels	Max(z)
dACC, vACC, vmPFC (bilateral)	Multiple regions	-2	40	0	501	0.00061
Anterior insula	AAIC L	-34	10	-8	17	0.00027
IFS	47l L	-38	28	-2	17	0.00035
Thalamus (bilateral)	Multiple regions	0	-10	8	271	0.00055
Caudate nucleus	Caudate Ca L	-12	14	6	25	0.00034
Ventral striatum	V Striatum L	0	24	8	11	0.00036
MT+	LO3 L	-48	-78	10	10	0.00023
STS	STSda L	-56	-16	-12	61	-0.00045
Inferior frontal cortex	p47r L	-38	48	2	49	-0.00079
PCC (incl. precuneus)	7m R	4	-60	34	484	-0.00073
IPC / TPJ	PGi R	52	-60	24	82	-0.00045
IPC / TPJ	PFm L	-56	-58	26	49	-0.00044
IPC / TPJ	PGs L	-34	-76	42	12	-0.00044
IPC / TPJ	PFm L	-40	-58	42	14	-0.00035

Note. Significant positive and negative weights contributing to the Self-vs-Other Referential Signature (SvO-RS). SVM classifiers were trained using 5000 bootstrap samples which produced two-tailed, uncorrected *p*-values. We applied an FDR-correction ($q < 0.05$ with cluster size $k > 10$) across the masked whole-brain to illustrate regions that contributed to the models most strongly. L refers to the left hemisphere, and R refers to the right hemisphere. Cortical atlas regions are labeled based on a combination of parcellations available on GitHub: https://github.com/canlab/Neuroimaging_Pattern_Masks/tree/master/Atlases_and_parcellations/2018_Wager_combined_atlas.

Table S6

Prediction results of the mentalizing signatures across training and validation datasets

Self-Mentalizing Signature (MS-Self)					
**Dataset**	**Study**	**Sample**	**n**	**Classification task**	**Prediction outcome**
Training	Study 1	Healthy adult	21	Self vs Other	acc.=1.00(+/-0.00), *P*<0.0001***, sens.=1.00, spec.=1.00, AUC=1.00, *d*=2.61
				Self vs Control	acc.=1.00(+/-0.00), *P*<0.0001***, sens.=1.00, spec.=1.00, AUC=1.00, *d*=3.74
Validation	Study 2	Adolescent	44	Self vs Other	acc.=0.77(+/-0.06), P=0.0004***, sens.=0.77, spec.=0.77, AUC=0.88, *d*=1.13
				Self vs Control	acc.=0.98(+/-0.02), *P*<0.0001***, sens.=0.98, spec.=0.98, AUC=1.00, *d*=2.60
Validation	Study 3	Adolescent	61	Self vs Other	acc.=0.75(+/-0.06), *P*<0.0001***, sens.=0.75, spec.=0.75, AUC=0.84, *d*=0.95
				Self vs Control	acc.=1.00(+/-0.00), *P*<0.0001***, sens.=1.00, spec.=1.00, AUC=1.00, *d*=3.52
Validation	Study 4a	Healthy adult	33	Self vs Other	acc.=0.91(+/-0.05), *P*<0.0001***, sens.=0.91, spec.=0.91, AUC=0.98, *d*=1.83
				Self vs Control	acc.=0.82(+/-0.07), *P*=0.0003***, sens.=0.82, spec.=0.82, AUC=0.93, *d*=1.51
Validation	Study 4b	Schizophrenia patient	23	Self vs Other	acc.=0.78(+/-0.09), *P*=0.0106***, sens.=0.78, spec.=0.78, AUC=0.71, *d*=0.50
				Self vs Control	acc.=0.83(+/-0.08), *P*=0.0026***, sens.=0.83, spec.=0.83, AUC=0.87, *d*=1.01
Validation	Study 5a	Healthy adult	15	Self vs Other	acc.=0.47(+/-0.13), *P*>0.20, sens.=0.47, spec.=0.47, AUC=0.71, *d*=0.61
				Self vs Control	acc.=0.53(+/-0.13), *P*>0.20, sens.=0.53, spec.=0.53, AUC=0.60, *d*=0.31
Validation	Study 5b	Schizophrenia patient	17	Self vs Other	acc.=0.76(+/-0.10), *P*=0.0490***, sens.=0.76, spec.=0.76, AUC=0.78, *d*=0.60
				Self vs Control	acc.=0.71(+/-0.11), *P*=0.1435, sens.=0.71, spec.=0.71, AUC=0.70, *d*=0.39
Validation	Study 5c	Bipolar patient	18	Self vs Other	acc.=0.78(+/-0.10), *P*=0.0309***, sens.=0.78, spec.=0.78, AUC=0.74, *d*=0.58
				Self vs Control	acc.=0.67(+/-0.11), *P*>0.20, sens.=0.67, spec.=0.67, AUC=0.71, *d*=0.35
Other-Mentalizing Signature (MS-Other)					
Signature	Dataset	Sample	n	Classification Task	Prediction Outcome
Training	Study 1	Healthy adult	21	Other vs Self	acc.=1.00(+/-0.00), *P*<0.0001***, sens.=1.00, spec.=1.00, AUC=1.00, *d*=2.36
				Other vs Control	acc.=1.00(+/-0.00), *P*<0.0001***, sens.=1.00, spec.=1.00, AUC=1.00, *d*=2.53
Validation	Study 2	Adolescent	44	Other vs Self	acc.=0.70(+/-0.07), *P*=0.0096***, sens.=0.70, spec.=0.70, AUC=0.83, *d*=0.90
				Other vs Control	acc.=0.95(+/-0.03), *P*<0.0001***, sens.=0.95, spec.=0.95, AUC=0.97, *d*=0.99
Validation	Study 3	Adolescent	61	Other vs Self	acc.=0.75(+/-0.06), *P*<0.0001***, sens.=0.75, spec.=0.75, AUC=0.80, *d*=0.81
				Other vs Control	acc.=0.38(+/-0.06), *P*>0.20, sens.=0.38, spec.=0.38, AUC=0.39, *d*=0.39
Validation	Study 4a	Healthy adult	33	Other vs Self	acc.=0.94(+/-0.04), *P*<0.0001***, sens.=0.94, spec.=0.94, AUC=0.99, *d*=2.15
				Other vs Control	acc.=0.88(+/-0.06), *P*<0.0001***, sens.=0.88, spec.=0.88, AUC=0.95, *d*=1.55

Validation	Study 4b	Schizophrenia patient	23	Other vs Self	acc.=0.70(+/-0.10), *P*=0.0931, sens.=0.70, spec.=0.70, AUC=0.81, *d*=0.83
				Other vs Control	acc.=0.83(+/-0.08), *P*=0.0026***, sens.=0.83, spec.=0.83, AUC=0.90, *d*=1.21
Validation	Study 5a	Healthy adult	15	Other vs Self	acc.=0.87(+/-0.09), *P*=0.0074***, sens.=0.87, spec.=0.87, AUC=0.97, *d*=1.65
				Other vs Control	acc.=1.00(+/-0.00), *P*<0.0001***, sens.=1.00, spec.=1.00, AUC=1.00, *d*=2.73
Validation	Study 5b	Schizophrenia patient	17	Other vs Self	acc.=0.76(+/-0.10), *P*=0.0490***, sens.=0.76, spec.=0.76, AUC=0.93 , *d*=1.34
				Other vs Control	acc.=0.94(+/-0.06), *P*=0.0003***, sens.=0.94, spec.=0.94, AUC=0.99, *d*=1.89
Validation	Study 5c	Bipolar patient	18	Other vs Self	acc.=0.83(+/-0.09), *P*=0.0075***, sens.=0.83, spec.=0.83, AUC=0.86, *d*=1.02
				Other vs Control	acc.=0.94(+/-0.05), *P*=0.0002***, sens.=0.94, spec.=0.94, AUC=0.96, *d*=1.79

Mentalizing Signature (MS)

Signature	Dataset	Sample	n	Classification Task	Prediction Outcome
Training	Study 1	Healthy adult	21	Self vs Control	acc.=1.00(+/-0.00), *P*<0.0001***, sens.=1.00, spec.=1.00, AUC=1.00, *d*=5.12
				Other vs Control	acc.=1.00(+/-0.00), *P*<0.0001***, sens.=1.00, spec.=1.00, AUC=1.00, *d*=4.72
Validation	Study 2	Adolescent	44	Self vs Control	acc.=1.00(+/-0.00), *P*<0.0001***, sens.=1.00, spec.=1.00, AUC=1.00, *d*=4.84
				Other vs Control	acc.=1.00(+/-0.00), *P*<0.0001***, sens.=1.00, spec.=1.00, AUC=1.00, *d*=4.99
Validation	Study 3	Adolescent	61	Self vs Control	acc.=1.00(+/-0.00), *P*<0.0001***, sens.=1.00, spec.=1.00, AUC=1.00, *d*=3.76
				Other vs Control	acc.=1.00(+/-0.00), *P*<0.0001***, sens.=1.00, spec.=1.00, AUC=1.00, *d*=3.98
Validation	Study 4a	Healthy adult	33	Self vs Control	acc.=0.97(+/-0.03), *P*<0.0001***, sens.=0.97, spec.=0.97, AUC=1.00, *d*=2.13
				Other vs Control	acc.=0.97(+/-0.03), *P*<0.0001***, sens.=0.97, spec.=0.97, AUC=0.98, *d*=1.87
Validation	Study 4b	Schizophrenia patient	23	Self vs Control	acc.=0.91(+/-0.06), *P*<0.0001***, sens.=0.91, spec.=0.91, AUC=0.99, *d*=1.61
				Other vs Control	acc.=0.96(+/-0.04), *P*<0.0001***, sens.=0.96, spec.=0.96, AUC=0.98, *d*=1.74
Validation	Study 5a	Healthy adult	15	Self vs Control	acc.=1.00(+/-0.00), *P*<0.0001***, sens.=1.00, spec.=1.00, AUC=1.00, *d*=2.47
				Other vs Control	acc.=1.00(+/-0.00), *P*<0.0001***, sens.=1.00, spec.=1.00, AUC=1.00, *d*=3.36
Validation	Study 5b	Schizophrenia patient	17	Self vs Control	acc.=1.00(+/-0.00), *P*<0.0001***, sens.=1.00, spec=1.00, AUC=1.00, *d*=1.95
				Other vs Control	acc.=0.94(+/-0.06), *P*=0.0003***, sens.=0.94, spec.=0.94, AUC=0.98, *d*=1.91
Validation	Study 5c	Bipolar patient	18	Self vs Control	acc.=0.89(+/-0.07), *P*=0.0013***, sens.=0.89, spec.=0.89, AUC=0.97, *d*=1.61
				Other vs Control	acc.=0.94(+/-0.05), *P*=0.0002***, sens.=0.94, spec.=0.94, AUC=0.99, *d*=2.19

Self-vs-Other Referential Signature (SvO-RS)

Signature	Dataset	Sample	n	Classification Task	Prediction Outcome
Training	Study 1	Healthy adult	21	Self vs Other	acc.=1.00(+/-0.00), *P*<0.0001***, sens.=1.00, spec.=1.00, AUC=1.00, *d*=2.45
Validation	Study 2	Adolescent	44	Self vs Other	acc.=0.73(+/-0.07), *P*=0.0037***, sens.=0.73, spec.=0.73, AUC=0.85, *d*=1.04

Validation	Study 3	Adolescent	61	Self vs Other	acc.=0.75(+/-0.06), *P*<0.0001***, sens.=0.75, spec.=0.75, AUC=0.80, *d*=0.82
Validation	Study 4a	Healthy adult	33	Self vs Other	acc.=0.91(+/-0.05), *P*<0.0001***, sens.=0.91, spec.=0.91, AUC=0.99, *d*=2.13
Validation	Study 4b	Schizophrenia patient	23	Self vs Other	acc.=0.70(+/-0.10), *P*=0.0931, sens.=0.70, spec.=0.70, AUC=0.78, *d*=0.70
Validation	Study 5a	Healthy adult	15	Self vs Other	acc.=0.80(+/-0.10), *P*=0.0352***, sens.=0.80, spec.=0.80, AUC=0.93, *d*=1.37
Validation	Study 5b	Schizophrenia patient	17	Self vs Other	acc.=0.76(+/-0.10), *P*=0.0490***, sens.=0.76, spec.=0.76, AUC=0.93, *d*=1.32
Validation	Study 5c	Bipolar patient	18	Self vs Other	acc.=0.83(+/-0.09), *P*=0.0075***, sens.=0.83, spec.=0.83, AUC=0.83, *d*=0.89

Note. The predictions of signatures were assigned using paired observations with a forced-choice principle. The signatures' classification accuracies were assessed using Receiver Operating Characteristic (ROC) analysis and binomial tests using a two-sided significance threshold of $p < .05$. acc. = accuracy; sens. = sensitivity; spec. = specificity, AUC = area under the curve. *d* refers to the estimated *Cohen's d* calculated as the mean difference of true and false paired predictions divided by the pooled standard deviation of differences (where difference = input_values[binary_class]- input_values[~binary_class]). Asterisks (***) mark the significant classification accuracies with $p < .05$. Prediction accuracies significantly above chance level (50%) at $p < .05$ are shown in black font, whereas accuracies not significantly above chance level are shown in grey font.

Supplementary Table 7

Prediction results of the ROI classifiers across training and validation datasets

ROI 1: mPFC			
**Trained for**	**Task**	**Sample**	**Prediction Outcome**
Predicting Self-mentalizing	Self vs Other	Training (*n* = 21)	acc.=0.95(+/-0.05), *P*<0.0001***, sens.=0.95, spec.=0.95, AUC=1.00, *d* =2.17
		Validation (*n* = 211)	acc.=0.66(+/-0.03), *P*<0.0001***, sens.=0.66, spec.=0.66, AUC=0.69, *d*=0.37
	Self vs Control	Training (*n* = 21)	acc.=1.00(+/-0.00), *P*<0.0001***, sens.=1.00, spec.=1.00, AUC=1.00, *d*=2.29
		Validation (*n* = 211)	acc.=0.79(+/-0.03), *P*<0.0001***, sens.=0.79, spec.=0.79, AUC=0.88, *d*=0.96
Predicting Other-mentalizing	Other vs Self	Training (*n* = 21)	acc.=1.00(+/-0.00), *P*<0.0001***, sens.=1.00, spec.=1.00, AUC=1.00, *d*=2.23
		Validation (*n* = 211)	acc.=0.63(+/-0.03), *P*=0.0002***, sens.=0.63, spec.=0.63, AUC=0.66, *d*=0.36
	Other vs Control	Training (*n* = 21)	acc.=0.86(+/-0.08), *P*=0.0015***, sens.=0.86, spec.=0.86, AUC=0.97, *d*=1.68
		Validation (*n* = 211)	acc.=0.78(+/-0.03), *P*<0.0001***, sens.=0.78, spec.=0.78, AUC=0.85, *d*=0.86
Predicting mentalizing	Self vs Control	Training (*n* = 21)	acc.=1.00(+/-0.00), *P*<0.0001***, sens.=1.00, spec.=1.00, AUC=1.00, *d*=5.63
		Validation (*n* = 211)	acc.=0.97(+/-0.01), *P*<0.0001***, sens.=0.97, spec.=0.97, AUC=0.99, *d*=1,64
	Other vs Control	Training (*n* = 21)	acc.=1.00(+/-0.00), *P*<0.0001***, sens.=1.00, spec.=1.00, AUC=1.00, *d*=6.01
		Validation (*n* = 211)	acc.=0.97(+/-0.01), *P*<0.0001***, sens.=0.97, spec.=0.97, AUC=0.99, *d*=1.69
Differentiating „self“ vs „other“ conditions	Self vs Other	Training (*n* = 21)	acc.=0.95(+/-0.05), *P*<0.0001***, sens.=0.95, spec.=0.95, AUC=1.00, *d*=2.10
		Validation (*n* = 211)	acc.=0.68(+/-0.03), *P*<0.0001***, sens.=0.68, spec.=0.68, AUC=0.68, *d*=0.36

ROI 2: Precuneus/PCC			
**Trained for**	**Task**	**Sample**	**Prediction Outcome**
Predicting Self-mentalizing	Self vs Other	Training (*n* = 21)	acc.=0.81(+/-0.09), *P*=0.0072***, sens.=0.81, spec.=0.81, AUC=0.90, *d*=1.20
		Validation (*n* = 211)	acc.=0.70(+/-0.03), *P*<0.0001***, sens.=0.70, spec.=0.70, AUC=0.74, *d*=0.57
	Self vs Control	Training (*n* = 21)	acc.=0.90(+/-0.06), *P*=0.0002***, sens.=0.90, spec.=0.90, AUC=0.96, *d*=1.52
		Validation (*n* = 211)	acc.=0.68(+/-0.03), *P*<0.0001***, sens.=0.68, spec.=0.68, AUC=0.73, *d*=0.59
Predicting Other-mentalizing	Other vs Self	Training (*n* = 21)	acc.=1.00(+/-0.00), *P*<0.0001***, sens.=1.00, spec.=1.00, AUC=1.00, *d*=2.10
		Validation (*n* = 211)	acc.=0.81(+/-0.03), *P*<0.0001***, sens.=0.81, spec.=0.81, AUC=0.84, *d*=0.85
	Other vs Control	Training (*n* = 21)	acc.=0.95(+/-0.05), *P*<0.0001***, sens.=0.95, spec.=0.95, AUC=1.00, *d*=2.72
		Validation (*n* = 211)	acc.=0.91(+/-0.02), *P*<0.0001***, sens.=0.91, spec.=0.91, AUC=0.96, *d*=1.45
Predicting mentalizing	Self vs Control	Training (*n* = 21)	acc.=1.00(+/-0.00), *P*<0.0001***, sens.=1.00, spec.=1.00, AUC=1.00, *d*=3.11
		Validation (*n* = 211)	acc.=0.91(+/-0.02), *P*<0.0001***, sens.=0.91, spec.=0.91, AUC=0.96, *d*=1.29
	Other vs Control	Training (*n* = 21)	acc.=1.00(+/-0.00), *P*<0.0001***, sens.=1.00, spec.=1.00, AUC=1.00, *d*=3.42

		Validation (*n* = 211)	acc.=0.91(+/-0.02), *P*<0.0001***, sens.=0.91, spec.=0.91, AUC=0.97, *d*=1.40
Differentiating „self" vs „other" conditions	Self vs Other	Training (*n* = 21)	acc.=1.00(+/-0.00), *P*<0.0001***, sens.=1.00, spec.=1.00, AUC=1.00, *d*=2.07
		Validation (*n* = 211)	acc.=0.75(+/-0.03), *P*<0.0001***, sens.=0.75, spec.=0.75, AUC=0.82, *d*=0.78

ROI 3: TPJ right

Trained for	Task	Sample	Prediction Outcome
Predicting Self-mentalizing	Self vs Other	Training (*n* = 21)	acc.=0.81(+/-0.09), *P*=0.0072***,sens.=0.81, spec.=0.81, AUC=0.84, *d*=0.69
		Validation (*n* = 211)	acc.=0.47(+/-0.03), *P*>0.20, sens.=0.47, spec.=0.47, AUC=0.49, *d*=0.05
	Self vs Control	Training (*n* = 21)	acc.=0.76(+/-0.09), *P*=0.0266***, sens.=0.76, spec.=0.76, AUC=0.88, *d*=1.03
		Validation (*n* = 211)	acc.=0.63(+/-0.03), *P*=0.0003***, sens.=0.63, spec.=0.63, AUC=0.69, *d*=0.45
Predicting Other-mentalizing	Other vs Self	Training (*n* = 21)	acc.=0.76(+/-0.09), *P*=0.0266***, sens.=0.76, spec.=0.76, AUC=0.86, *d*=0.90
		Validation (*n* = 211)	acc.=0.69(+/-0.03), *P*<0.0001***, sens.=0.69, spec.=0.69, AUC=0.74, *d*=0.49
	Other vs Control	Training (*n* = 21)	acc.=0.81(+/-0.09), *P*=0.0072***, sens.=0.81, spec.=0.81, AUC=0.92, *d*=1.12
		Validation (*n* = 211)	acc.=0.69(+/-0.03), *P*<0.0001***, sens.=0.69, spec.=0.69, AUC=0.74, *d*=0.60
Predicting mentalizing	Self vs Control	Training (*n* = 21)	acc.=1.00(+/-0.00), *P*<0.0001***, sens.=1.00, spec.=1.00, AUC=1.00, *d*=3.32
		Validation (*n* = 211)	acc.=0.80(+/-0.03), *P*<0.0001***, sens.=0.80, spec.=0.80, AUC=0.90, *d*=1.07
	Other vs Control	Training (*n* = 21)	acc.=1.00(+/-0.00), *P*<0.0001***, sens.=1.00, spec.=1.00, AUC=1.00, *d*=2.55
		Validation (*n* = 211)	acc.=0.86(+/-0.02), *P*<0.0001***, sens.=0.86, spec.=0.86, AUC=0.93, *d*=1.15
Differentiating „self" vs „other" conditions	Self vs Other	Training (*n* = 21)	acc.=0.76(+/-0.09), *P*=0.0266***, sens.=0.76, spec.=0.76, AUC=0.84, *d*=0.82
		Validation (*n* = 211)	acc.=0.65(+/-0.03), *P*<0.0001***, sens.=0.65, spec.=0.65, AUC=0.71, *d*=0.45

ROI 4: TPJ left

Trained for	Task	Sample	Prediction Outcome
Predicting Self-mentalizing	Self vs Other	Training (*n* = 21)	acc.=0.57(+/-0.11), *P*>0.20, sens.=0.57, spec.=0.57, AUC=0.64, *d*=0.23
		Validation (*n* = 211)	acc.=0.56(+/-0.03), *P*=0.0983, sens.=0.56, spec.=0.56, AUC=0.60, *d*=0.24
	Self vs Control	Training (*n* = 21)	acc.=0.81(+/-0.09), *P*=0.0072***, sens.=0.81, spec.=0.81, AUC=0.75, *d*=0.57
		Validation (*n* = 211)	acc.=0.69(+/-0.03), *P*<0.0001***, sens.=0.69, spec.=0.69, AUC=0.80, *d*=0.74
Predicting Other-mentalizing	Other vs Self	Training (*n* = 21)	acc.=0.81(+/-0.09), *P*=0.0072***, sens.=0.81, spec.=0.81, AUC=0.87, *d*=0.95
		Validation (*n* = 211)	acc.=0.71(+/-0.03), *P*<0.0001***, sens.=0.71, spec.=0.71, AUC=0.72, *d*=0.44
	Other vs Control	Training (*n* = 21)	acc.=0.90(+/-0.06), *P*=0.0002***, sens.=0.90, spec.=0.90, AUC=0.98, *d*=1.81
		Validation (*n* = 211)	acc.=0.81(+/-0.03), *P*<0.0001***, sens.=0.81, spec.=0.81, AUC=0.90, *d*=1.07
Predicting mentalizing	Self vs Control	Training (*n* = 21)	acc.=1.00(+/-0.00), *P*<0.0001***, sens.=1.00, spec.=1.00, AUC=1.00, *d*=3.04

		Validation (*n* = 211)	acc.=0.89(+/-0.02), *P*<0.0001***, sens.=0.89, spec.=0.89, AUC=0.96, *d*=1.19
	Other vs Control	Training (*n* = 21)	acc.=1.00(+/-0.00), *P*<0.0001***, sens.=1.00, spec.=1.00, AUC=1.00, *d*=2.70
		Validation (*n* = 211)	acc.=0.92(+/-0.02), *P*<0.0001***, sens.=0.92, spec.=0.92, AUC=0.98, *d*=1.23
Differentiating „self“ vs „other“ conditions	Self vs Other	Training (*n* = 21)	acc.=0.86(+/-0.08), *P*=0.0015***, sens.=0.86, spec.=0.86, AUC=0.88, *d*=1.04
		Validation (*n* = 211)	acc.=0.68(+/-0.03), *P*<0.0001***, sens.=0.68, spec.=0.68, AUC=0.72, *d*=0.45

ROI 5: aMTG right

**Trained for**	**Task**	**Sample**	**Prediction Outcome**
Predicting Self-mentalizing	Self vs Other	Training (*n* = 21)	acc.=0.67(+/-0.10), *P*=0.1893, sens.=0.67, spec.=0.67, AUC=0.74, *d*=0.51
		Validation (*n* = 211)	acc.=0.64(+/-0.03), *P*<0.0001***, sens.=0.64, spec.=0.64, AUC=0.69, *d*=0.47
	Self vs Control	Training (*n* = 21)	acc.=0.67(+/-0.10), *P*=0.1893, sens.=0.67, spec.=0.67, AUC=0.75, *d*=0.59
		Validation (*n* = 211)	acc.=0.41(+/-0.03), *P*>0.20, sens.=0.41, spec.=0.41, AUC=0.36, *d*=-0.37
Predicting Other-mentalizing	Other vs Self	Training (*n* = 21)	acc.=0.81(+/-0.09), *P*=0.0072***, sens.=0.81, spec.=0.81, AUC=0.86, *d*=1.11
		Validation (*n* = 211)	acc.=0.66(+/-0.03), *P*<0.0001***, sens.=0.66, spec.=0.66, AUC=0.69, *d*=0.46
	Other vs Control	Training (*n* = 21)	acc.=0.81(+/-0.09), *P*=0.0072***, sens.=0.81, spec.=0.81, AUC=0.94, *d*=1.35
		Validation (*n* = 211)	acc.=0.84(+/-0.03), *P*<0.0001***, sens.=0.84, spec.=0.84, AUC=0.92, *d*=1.13
Predicting mentalizing	Self vs Control	Training (*n* = 21)	acc.=0.76(+/-0.09), *P*=0.0266***, sens.=0.76, spec.=0.76, AUC=0.92, *d*=1.25
		Validation (*n* = 211)	acc.=0.80(+/-0.03), *P*<0.0001***, sens.=0.80, spec.=0.80, AUC=0.87, *d*=1.00
	Other vs Control	Training (*n* = 21)	acc.=0.86(+/-0.08), *P*=0.0015***, sens.=0.86, spec.=0.86, AUC=0.95, *d*=1.39
		Validation (*n* = 211)	acc.=0.83(+/-0.03), *P*<0.0001***, sens.=0.83, spec.=0.83, AUC=0.89, *d*=1.00
Differentiating „self“ vs „other“ conditions	Self vs Other	Training (*n* = 21)	acc.=0.81(+/-0.09), *P*=0.0072***, sens.=0.81, spec.=0.81, AUC=0.84, *d*=0.90
		Validation (*n* = 211)	acc.=0.65(+/-0.03), *P*<0.0001***, sens.=0.65, spec.=0.65, AUC=0.71, *d*=0.51

ROI 6: aMTG left

**Trained for**	**Task**	**Sample**	**Prediction Outcome**
Predicting Self-mentalizing	Self vs Other	Training (*n* = 21)	acc.=0.95(+/-0.05), *P*<0.0001***, sens.=0.95, spec.=0.95, AUC=0.98, *d*=1.83
		Validation (*n* = 211)	acc.=0.57(+/-0.03), *P*=0.0386***, sens.=0.57, spec.=0.57, AUC=0.63, *d*=0.35
	Self vs Control	Training (*n* = 21)	acc.=1.00(+/-0.00), *P*<0.0001***, sens.=1.00, spec.=1.00, AUC=1.00, *d*=2.53
		Validation (*n* = 211)	acc.=0.75(+/-0.03), *P*<0.0001***, sens.=0.75, spec.=0.75, AUC=0.81, *d*=0.87
Predicting Other-mentalizing	Other vs Self	Training (*n* = 21)	acc.=0.86(+/-0.08), *P*=0.0015***, sens.=0.86, spec.=0.86, AUC=0.96, *d*=1.69
		Validation (*n* = 211)	acc.=0.71(+/-0.03), *P*<0.0001***, sens.=0.71, spec.=0.71, AUC=0.80, *d*=0.81
	Other vs Control	Training (*n* = 21)	acc.=0.95(+/-0.05), *P*<0.0001***, sens.=0.95, spec.=0.95, AUC=0.97, *d*=1.71
		Validation (*n* = 211)	acc.=0.73(+/-0.03), *P*<0.0001***, sens.=0.73,

			spec.=0.73, AUC=0.80, *d*=0.74
Predicting mentalizing	Self vs Control	Training (*n* = 21)	acc.=1.00(+/-0.00), *P*<0.0001***, sens.=1.00, spec.=1.00, AUC=1.00, *d*=4.43
		Validation (*n* = 211)	acc.=0.92(+/-0.02), *P*<0.0001***, sens.=0.92, spec.=0.92, AUC=0.97, *d*=1.30
	Other vs Control	Training (*n* = 21)	acc.=1.00(+/-0.00), *P*<0.0001***, sens.=1.00, spec.=1.00, AUC=1.00, *d*=3.49
		Validation (*n* = 211)	acc.=0.94(+/-0.02), *P*<0.0001***, sens.=0.94, spec.=0.94, AUC=0.98, *d*=1.47
Differentiating „self“ vs „other“ conditions	Self vs Other	Training (*n* = 21)	acc.=0.95(+/-0.05), *P*<0.0001***, sens.=0.95, spec.=0.95, AUC=0.99, *d*=1.91
		Validation (*n* = 211)	acc.=0.64(+/-0.03), *P*<0.0001***, sens.=0.64, spec.=0.64, AUC=0.69, *d*=0.49
ROI 7: SMA left			
**Trained for**	**Task**	**Sample**	**Prediction Outcome**
Predicting Self-mentalizing	Self vs Other	Training (*n* = 21)	acc.=0.67(+/-0.10), *P*=0.1893, sens.=0.67, spec.=0.67, AUC=0.70, *d*=0.23
		Validation (*n* = 211)	acc.=0.52(+/-0.03), *P*>0.20, sens.=0.52, spec.=0.52, AUC=0.51, *d*=-0.02
	Self vs Control	Training (*n* = 21)	acc.=1.00(+/-0.00), *P*<0.0001***, sens.=1.00, spec.=1.00, AUC=1.00, *d*=2.44
		Validation (*n* = 211)	acc.=0.48(+/-0.03), *P*>0.20, sens.=0.48, spec.=0.48, AUC=0.53, *d*=0.12
Predicting Other-mentalizing	Other vs Self	Training (*n* = 21)	acc.=0.43(+/-0.11), *P*>0.20, sens.=0.43, spec.=0.43, AUC=0.51, *d*=-0.02
		Validation (*n* = 211)	acc.=0.50(+/-0.03), *P*>0.20, sens.=0.50, spec.=0.50, AUC=0.51, *d*=-0.08
	Other vs Control	Training (*n* = 21)	acc.=0.62(+/-0.11), *P*>0.20, sens.=0.62, spec.=0.62, AUC=0.81, *d*=0.87
		Validation (*n* = 211)	acc.=0.71(+/-0.03), *P*<0.0001***, sens.=0.71, spec.=0.71, AUC=0.75, *d*=0.60
Predicting mentalizing	Self vs Control	Training (*n* = 21)	acc.=1.00(+/-0.00), *P*<0.0001***, sens.=1.00, spec.=1.00, AUC=1.00, *d*=4.30
		Validation (*n* = 211)	acc.=0.85(+/-0.02), *P*<0.0001***, sens.=0.85, spec.=0.85, AUC=0.94, *d*=1.20
	Other vs Control	Training (*n* = 21)	acc.=0.95(+/-0.05), *P*<0.0001***, sens.=0.95, spec.=0.95, AUC=1.00, *d*=3.05
		Validation (*n* = 211)	acc.=0.86(+/-0.02), *P*<0.0001***, sens.=0.86, spec.=0.86, AUC=0.93, *d*=1.20
Differentiating „self“ vs „other“ conditions	Self vs Other	Training (*n* = 21)	acc.=0.62(+/-0.11), *P*>0.20, sens.=0.62, spec.=0.62, AUC=0.69, *d*=0.20
		Validation (*n* = 211)	acc.=0.50(+/-0.03), *P*>0.20, sens.=0.50, spec.=0.50, AUC=0.50, *d*=-0.03
ROI 8: Cerebellum bilateral			
**Trained for**	**Task**	**Sample**	**Prediction Outcome**
Predicting Self-mentalizing	Self vs Other	Training (*n* = 21)	acc.=0.62(+/-0.11), *P*>0.20, sens.=0.62, spec.=0.62, AUC=0.65, *d*=0.29
		Validation (*n* = 211)	acc.=0.52(+/-0.03), *P*>0.20, sens.=0.52, spec=0.52, AUC=0.56, *d*=0.10
	Self vs Control	Training (*n* = 21)	acc.=0.86(+/-0.08), *P*=0.0015***, sens.=0.86, spec.=0.86, AUC=0.94, *d*=1.48
		Validation (*n* = 211)	acc.=0.67(+/-0.03), *P*<0.0001***, sens.=0.67, spec.=0.67, AUC=0.71, *d*=0.39
Predicting Other-mentalizing	Other vs Self	Training (*n* = 21)	acc.=0.48(+/-0.11), *P*>0.20, sens.=0.48, spec.=0.48, AUC=0.54, *d*=0.06
		Validation (*n* = 211)	acc.=0.54(+/-0.03), *P*>0.20, sens.=0.54, spec.=0.54, AUC=0.55, *d*=0.05

Trained for	Task	Sample	Prediction Outcome
	Other vs Control	Training (*n* = 21)	acc.=0.76(+/-0.09), *P*=0.0266***, sens.=0.76, spec.=0.76, AUC=0.85, *d*=0.81
		Validation (*n* = 211)	acc.=0.45(+/-0.03), *P*>0.20, sens.=0.45, spec.=0.45, AUC=0.46, *d*=-0.10
Predicting mentalizing	Self vs Control	Training (*n* = 21)	acc.=1.00(+/-0.00), *P*<0.0001***, sens.=1.00, spec.=1.00, AUC=1.00, *d*=2.70
		Validation (*n* = 211)	acc.=0.69(+/-0.03), *P*<0.0001***, sens.=0.69, spec.=0.69, AUC=0.76, *d*=0.61
	Other vs Control	Training (*n* = 21)	acc.=0.95(+/-0.05), *P*<0.0001***, sens.=0.95, spec.=0.95, AUC=1.00, *d*=2.61
		Validation (*n* = 211)	acc.=0.73(+/-0.03), *P*<0.0001***, sens.=0.73, spec.=0.73, AUC=0.77, *d*=0.68
Differentiating „self“ vs „other“ conditions	Self vs Other	Training (*n* = 21)	acc.=0.62(+/-0.11), *P*>0.20, sens.=0.62, spec.=0.62, AUC=0.64, *d*=0.26
		Validation (*n* = 211)	acc.=0.54(+/-0.03), *P*>0.20, sens.=0.54, spec.=0.54, AUC=0.56, *d*=0.07

ROI 9: Cerebellum right

Trained for	Task	Sample	Prediction Outcome
Predicting Self-mentalizing	Self vs Other	Training (*n* = 21)	acc.=0.48(+/-0.11), *P*>0.20, sens.=0.48, spec.=0.48, AUC=0.46, *d*=-0.31
		Validation (*n* = 211)	acc.=0.40(+/-0.03), *P*>0.20, sens.=0.40, spec.=0.40, AUC=0.37, *d*=-0.34
	Self vs Control	Training (*n* = 21)	acc.=0.76(+/-0.09), *P*=0.0266***, sens.=0.76, spec.=0.76, AUC=0.84, *d*=0.95
		Validation (*n* = 211)	acc.=0.73(+/-0.03), *P*<0.0001***, sens.=0.73, spec.=0.73, AUC=0.81, *d*=0.79
Predicting Other-mentalizing	Other vs Self	Training (*n* = 21)	acc.=0.67(+/-0.10), *P*=0.1893, sens.=0.67, spec.=0.67, AUC=0.62, *d*=0.21
		Validation (*n* = 211)	acc.=0.49(+/-0.03), *P*>0.20, sens.=0.49, spec.=0.49, AUC=0.47, *d*=-0.10
	Other vs Control	Training (*n* = 21)	acc.=0.90(+/-0.06), *P*=0.0002***, sens.=0.90, spec.=0.90, AUC=0.95, *d*=1.31
		Validation (*n* = 211)	acc.=0.60(+/-0.03), *P*=0.0058***, sens.=0.60, spec.=0.60, AUC=0.64, *d*=0.38
Predicting mentalizing	Self vs Control	Training (*n* = 21)	acc.=1.00(+/-0.00), *P*<0.0001***, sens.=1.00, spec.=1.00, AUC=1.00, *d*=3.52
		Validation (*n* = 211)	acc.=0.88(+/-0.02), *P*<0.0001***, sens.=0.88, spec.=0.88, AUC=0.96, *d*=1.32
	Other vs Control	Training (*n* = 21)	acc.=1.00(+/-0.00), *P*<0.0001***, sens.=1.00, spec.=1.00, AUC=1.00, *d*=3.61
		Validation (*n* = 211)	acc.=0.88(+/-0.02), *P*<0.0001***, sens.=0.88, spec.=0.88, AUC=0.95, *d*=1.36
Differentiating „self“ vs „other“ conditions	Self vs Other	Training (*n* = 21)	acc.=0.57(+/-0.11), *P*>0.20, sens.=0.57, spec.=0.57, AUC=0.51, *d*=-0.14
		Validation (*n* = 211)	acc.=0.43(+/-0.03), *P*>0.20, sens.=0.43, spec.=0.43, AUC=0.41, *d*=-0.24

ROI 10: Cerebellum left

Trained for	Task	Sample	Prediction Outcome
Predicting Self-mentalizing	Self vs Other	Training (*n* = 21)	acc.=0.29(+/-0.10), *P*>0.20, sens.=0.29, spec.=0.29, AUC=0.45, *d*=-0.05
		Validation (*n* = 211)	acc.=0.49(+/-0.03), *P*>0.20, sens.=0.49, spec.=0.49, AUC=0.50, *d*=-0.02
	Self vs Control	Training (*n* = 21)	acc.=0.76(+/-0.09), *P*=0.0266***, sens.=0.76, spec.=0.76, AUC=0.90, *d*=0.94
		Validation (*n* = 211)	acc.=0.71(+/-0.03), *P*<0.0001***, sens.=0.71, spec.=0.71, AUC=0.78, *d*=0.73

Predicting Other-mentalizing	Other vs Self	Training (*n* = 21)	acc.=0.62(+/-0.11), *P*>0.20, sens.=0.62, spec.=0.62, AUC=0.63, *d*=0.12
		Validation (*n* = 211)	acc.=0.52(+/-0.03), *P*>0.20, sens.=0.52, spec.=0.52, AUC=0.50, *d*=-0.02
	Other vs Control	Training (*n* = 21)	acc.=0.81(+/-0.09), *P*=0.0072***, sens.=0.81, spec.=0.81, AUC=0.88, *d*=1.09
		Validation (*n* = 211)	acc.=0.62(+/-0.03), *P*=0.0006***, sens.=0.62, spec.=0.62, AUC=0.64, *d*=0.35
Predicting mentalizing	Self vs Control	Training (*n* = 21)	acc.=0.90(+/-0.06), *P*=0.0002***, sens.=0.90, spec.=0.90, AUC=0.99, *d*=2.26
		Validation (*n* = 211)	acc.=0.85(+/-0.02), *P*<0.0001***, sens.=0.85, spec.=0.85, AUC=0.93, *d*=1.12
	Other vs Control	Training (*n* = 21)	acc.=0.90(+/-0.06), *P*=0.0002***, sens.=0.90, spec.=0.90, AUC=0.99, *d*=2.10
		Validation (*n* = 211)	acc.=0.86(+/-0.02), *P*<0.0001***, sens.=0.86, spec.=0.86, AUC=0.93, *d*=1.08
Differentiating „self“ vs „other“ conditions	Self vs Other	Training (*n* = 21)	acc.=0.48(+/-0.11), *P*>0.20, sens.=0.48, spec.=0.48, AUC=0.58, *d*=0.11
		Validation (*n* = 211)	acc.=0.48(+/-0.03), *P*>0.20, sens.=0.48, spec.=0.48, AUC=0.49, *d*=-0.04

Note. Each ROI was trained for four classification tasks and tested in validation datasets. The signatures' classification accuracies were assessed using Receiver Operating Characteristic (ROC) analysis and binomial tests using a two-sided significance threshold of $p < .05$. acc. = accuracy; sens. = sensitivity, spec. = specificity, AUC = area under the curve. *d* refers to the estimated *Cohen's d* calculated as the mean difference of true and false paired predictions divided by the pooled standard deviation of differences (where difference = input_values[binary_class]- input_values[~binary_class]). Asterisks (***) mark the significant classification accuracies with $p < .05$. Prediction accuracies significantly above chance level (50%) at $p < .05$ are shown in black font, whereas accuracies not significantly above chance level are shown in grey font.
