# Supplementary material for: Brain neuromarkers predict self- and other-related mentalizing across adult, clinical, and developmental samples
Source: Nat Commun. 2026 Jun 6;17:7229. doi: 10.1038/s41467-026-73945-w (PMC13396484; doi:10.1038/s41467-026-73945-w)
Supplement: Supplementary file 2 — Reporting Summary [file 41467_2026_73945_MOESM2_ESM.pdf]

Corresponding author(s): Leonie Koban

Last updated by author(s): Apr 10, 2026

Reporting Summary

Nature Portfolio wishes to improve the reproducibility of the work that we publish. This form provides structure for consistency and transparency in reporting. For further information on Nature Portfolio policies, see our Editorial Policies and the Editorial Policy Checklist.

Statistics

For all statistical analyses, confirm that the following items are present in the figure legend, table legend, main text, or Methods section.

n/a	Confirmed	
☐	☒	The exact sample size (n) for each experimental group/condition, given as a discrete number and unit of measurement
☐	☒	A statement on whether measurements were taken from distinct samples or whether the same sample was measured repeatedly
☐	☒	The statistical test(s) used AND whether they are one- or two-sided<br>*Only common tests should be described solely by name; describe more complex techniques in the Methods section.*
☐	☒	A description of all covariates tested
☐	☒	A description of any assumptions or corrections, such as tests of normality and adjustment for multiple comparisons
☐	☒	A full description of the statistical parameters including central tendency (e.g. means) or other basic estimates (e.g. regression coefficient) AND variation (e.g. standard deviation) or associated estimates of uncertainty (e.g. confidence intervals)
☐	☒	For null hypothesis testing, the test statistic (e.g. F, t, r) with confidence intervals, effect sizes, degrees of freedom and P value noted<br>*Give P values as exact values whenever suitable.*
☒	☐	For Bayesian analysis, information on the choice of priors and Markov chain Monte Carlo settings
☐	☒	For hierarchical and complex designs, identification of the appropriate level for tests and full reporting of outcomes
☐	☒	Estimates of effect sizes (e.g. Cohen's d, Pearson's r), indicating how they were calculated

Our web collection on statistics for biologists contains articles on many of the points above.

Software and code

Policy information about availability of computer code

Data collection	Experimental tasks were presented using E-Prime 2.0 (Study 1, Study 2, Study 5) , Presentation 19.0 (Study 3), custom experimental program written in Tcl (Study 4), Psychophysics Toolbox 3.0.13–3.0.16 (Study 6), and PsychoPy 3.1.2 (Study 7)
Data analysis	Matlab 2022b; custom code available on https://github.com/ldmk/2025_MentalizingSignatures

For manuscripts utilizing custom algorithms or software that are central to the research but not yet described in published literature, software must be made available to editors and reviewers. We strongly encourage code deposition in a community repository (e.g. GitHub). See the Nature Portfolio guidelines for submitting code & software for further information.

Data

Policy information about availability of data

All manuscripts must include a data availability statement. This statement should provide the following information, where applicable:

- Accession codes, unique identifiers, or web links for publicly available datasets
- A description of any restrictions on data availability
- For clinical datasets or third party data, please ensure that the statement adheres to our policy

Source data for all figures and single-subject contrast images of the training dataset (Study 1) are available at94 https://doi.org/10.6084/m9.figshare.29908139. Contrast images from Study 4a can be accessed via OpenNeuro (https://openneuro.org/datasets/ds001618/versions/1.0.1) and data from Study 6 are available through the NIHM Archive (https://nda.nih.gov/edit_collection.html?id=2643). Imaging data from five contributing studies (Studies 2, 3, 4b, 5, and 7) are not shared online for privacy reasons; however, deidentified data from these studies can be made available in line with applicable data protection and privacy regulations upon

reasonable request to the corresponding authors.

Research involving human participants, their data, or biological material

Policy information about studies with human participants or human data. See also policy information about sex, gender (identity/presentation), and sexual orientation and race, ethnicity and racism.

Reporting on sex and gender	All studies recruited both male and female participants. Four studies (Study 1, 2, 4, 5, 6) collected information only on sex. Study 3 collected information also on gender. Study 7 collected information on gender and sexual orientation as well. Overall, the current study included comparable number of female (n=174) and male (n=216) participants. We did not have any hypotheses regarding sex or gender. We tested for sex differences in the pattern expression values as the main outcome variable.
Reporting on race, ethnicity, or other socially relevant groupings	Race and ethnicity information was only collected in Study 6 and 7 which were conducted in the USA. As a common practice in the European continent, the other studies did not collect this information. Therefore, we could not consider race, ethnicity, or other socially relevant groupings in our analyses. We acknowledge that mentalizing-related brain activity may show variations across culturally-divergent social groups. An advantage of the brain signatures approach is that the models are shared with the scientific community and their validity can be tested in different samples and cultural contexts in future studies.
Population characteristics	The training sample (Study 1; Koban et al., 2014) consisted of n=21 healthy adults (10 women and 11 men, Mean age = 23.7) who were recruited at the University of Geneva, Switzerland.<br><br>Study 2 (Debbane et al., 2017) included n=44 healthy adolescents (23 females and 21 males, Mean age = 16, SD age = 1.86, Age range = 12.01 to 18.84) who were recruited from secondary schools in Geneva, Switzerland.<br><br>Study 3 (van Buuren et al., 2020) included n=61 healthy adolescents (27 females and 34 males, Mean age = 12.9, SD age = 0.43, Age range = 11.61 to 14.22) who were recruited for a longitudinal project from secondary schools in the Netherlands.<br><br>Study 4 (Fuentes-Claramonte et al., 2019; Fuentes-Claramonte et al., 2020) included n=33 healthy adults (14 women and 19 men, Mean age = 41.7) and n=23 adults with schizophrenia (7 women and 16 men, Mean age = 37). The two cohorts were matched on age, sex, and a measure of general intelligence. The patients were recruited from a local psychiatric hospital in Barcelona, Spain based on diagnostic interviews. The schizophrenia diagnosis was confirmed using the Structured Clinical Interview for DSM Disorders (SCID; First, 2015).<br><br>Study 5 (Zhang et al., 2015) included three types of cohorts: Healthy adults (n=15, 6 women and 9 men, Mage = 33.3), adults with schizophrenia (SZ, n=17, 6 women and 11 men, Mage = 35.5), and adults with bipolar disorder (BD, n=18, 10 women and 8 men, Mage = 40.3). The clinical cohorts were recruited from several mental care institutions in the north of the Netherlands. The diagnoses of the patients were confirmed using the Mini International Neuropsychiatric Interview-Plus 5.0.0 (MINI-Plus; Sheehan et al., 1998). All BD patients were chosen among those who had a history of at least one psychotic episode. All three cohorts were matched with one another on age, sex, level of education, and a measure of general intelligence. The SZ and BP patients were additionally matched on the level of cognitive and clinical insight as measured by the Schedule of Assessment of Insight-Expanded version (SAI-E, clinical insight; Kemp & David, 1997) and the Beck Cognitive Insight Scale (BCIS, cognitive insight; Beck et al., 2004).<br><br>Study 6a (Tusche et al., 2023) included 59 healthy adults (26 women and 33 men; Mage= 28.3), and Study 6b included 50 (19 women and 31 men; Mage= 33.6) healthy adults from the Los Angeles metropolitan area; these final sample sizes reflect exclusions described below. One participant from each study was excluded due to poor task performance (>70% missed trials), and four additional participants from Study 6b were excluded due to outlier behavioral scores (>3 SDs from the mean, n=1) and excessive scanner motion (n=3). All participants were right-handed, had normal or corrected-to-normal vision, spoke English fluently, and had IQs in the normal range (assessed via the WASI-II).<br><br>Study 7 (Ma et al., 2024) included n=49 (26 women and 23 men, Mage = 22.6) healthy adults in romantic relationships recruited from the Tucson, Arizona community and surrounding areas. Community members were eligible to participate if they had been in a romantic relationship for at least six months, had no contraindications for MRI scanning, and did not meet criteria for active psychosis or mania at the time of screening. Both members of the couple completed all components of the study including the social feedback fMRI task.<br><br>All participants gave written informed consent and were compensated for their participation via monetary means or gifts.
Recruitment	The participants of Study 1 were recruited using flyers and mailing lists at the University of Geneva, Switzerland. n=21 participants were recruited in the original study. One additional participant with structural abnormalities in the brain was excluded from the original study and the present analysis.<br><br>Participants of Study 2 were recruited from secondary schools in Geneva, Switzerland by written advertisements circulated in schools and public areas. n=44 adolescents were recruited in the original study and are included in the current study. In the original study, one additional subject was excluded due to structural abnormalities in the brain, three due to incompletion of the paradigm, one due to signs of substance use, and five due to excessive movement.<br><br>Study 3 was part of a larger longitudinal study (n=692 participants) from secondary schools in the Netherlands. All participants who, during the first assessment, indicated willingness to participate in a subsequent MRI assessment were contacted to participate in Study 3. Eventually, n=66 healthy adolescents were recruited in Study 3. Data of 61 adolescents were included in the current study because 5 participants did not provide explicit consent for data sharing. An additional 18

	participants were excluded from the original study due to excessive movement, incorrect task completion, or measurement errors.<br><br>In Study 4, n=33 healthy adults were recruited non-medical staff working in the hospital, their acquaintances, and other sources from the community. n=23 adults with schizophrenia were recruited from a local psychiatric hospital in Barcelona, Spain based on diagnostic interviews. The schizophrenia diagnosis was confirmed using the Structured Clinical Interview for DSM Disorders (SCID; First, 2015).<br><br>In Study 5, n=18 adults with bipolar disorder (BD) were recruited from several mental care institutions in the north of the Netherlands. BD patients were chosen among those who had a history of at least one psychotic episode. Based on the BD cohort, subsamples of healthy adults (n=21) and participants with schizophrenia (SZ, n=17, 6 women and 11 men, Mage = 35.5) that took part in an earlier study (van Buuren et al., 2013) were selected based on the matching criteria. All three cohorts were matched with one another on age, sex, level of education, and a measure of general intelligence. The SZ and BP patients were also matched on the level of cognitive and clinical insight as measured by the Schedule of Assessment of Insight-Expanded version (SAI-E, clinical insight; Kemp & David, 1997) and the Beck Cognitive Insight Scale (BCIS, cognitive insight; Beck et al., 2004). Because the data of n=6 healthy adults were inaccessible at the time of the current study, only n=15 healthy adults were included in the current study.<br><br>Study 6a (Tusche et al., 2023) included 59 healthy adults and Study 6b included 50 healthy adults recruited from the Los Angeles metropolitan area. Participants were e-mailed for participation who were part of a database at the time, as they had been recruited for other projects before.<br><br>Study 7 included n=56 healthy adults in monogamous romantic relationships recruited from the Tucson, Arizona community and surrounding areas using flyers and social media advertisements. Seven participants were excluded from the study due to missing or inadequate imaging data, yielding a final sample size of n=49.<br><br>All participants gave written informed consent and were compensated for their participation via monetary means or gifts.
Ethics oversight	All studies were approved by the respective institutional ethics committees (Study 1: Institutional Review Board of the University of Geneva; Study 2: Institutional Review Board of the Department of Psychiatry of the University of Medicine, Geneva; Study 3: Institutional Review Board of the Faculty of Behavioral and Movement Sciences, VU Amsterdam; Study 4: the Research Ethics Committee of FIDMAG Sisters Hospitals (Comité de Ética de la Investigación de FIDMAG Hermanas Hospitalarias); Study 5: Medical Ethics Committee of the University Medical Center Groningen; Study 6: Institutional Review Board of the California Institute of Technology; Study 7: Institutional Review Board the University of Arizona)

Note that full information on the approval of the study protocol must also be provided in the manuscript.

Field-specific reporting

Please select the one below that is the best fit for your research. If you are not sure, read the appropriate sections before making your selection.

[x] Life sciences [ ] Behavioural & social sciences [ ] Ecological, evolutionary & environmental sciences

For a reference copy of the document with all sections, see nature.com/documents/nr-reporting-summary-flat.pdf

Life sciences study design

All studies must disclose on these points even when the disclosure is negative.

Sample size	The study combined data (N=390) from seven independent studies. Therefore, the sample size (N=390) was based on the available data and is comparable or greater than previous work that used a similar approach in other domains (e.g., Wager et al., 2013; Koban et al., 2023)
Data exclusions	No data was excluded in this study after combining independent datasets. The original studies had their own inclusion criteria that are explained in the methods section and above under Recruitment.
Replication	Mentalizing signatures generalized to six independent validation datasets with different sample characteristics. The signatures (classifiers) are available for future studies to test further generalizibility in different contexts.
Randomization	The experimental tasks used in each study involved randomization of the order of (within-subject) experimental conditions, blocks, and/or stimuli. Randomization of study groups (adults with and without clinical diagnosis) was not possible.
Blinding	There were no experimental groups in this study; all experiments used a within subject design to test the primary task effects. Additionally, only two studies (Study 4 and 5) included mixed designs with participants from different groups. Neither of these two studies included a blinding procedure.

Reporting for specific materials, systems and methods

We require information from authors about some types of materials, experimental systems and methods used in many studies. Here, indicate whether each material, system or method listed is relevant to your study. If you are not sure if a list item applies to your research, read the appropriate section before selecting a response.

Materials & experimental systems

n/a	Involved in the study
x	Antibodies
x	Eukaryotic cell lines
x	Palaeontology and archaeology
x	Animals and other organisms
x	Clinical data
x	Dual use research of concern
x	Plants

Methods

n/a	Involved in the study
x	ChIP-seq
x	Flow cytometry
	x MRI-based neuroimaging

Plants

Seed stocks

Report on the source of all seed stocks or other plant material used. If applicable, state the seed stock centre and catalogue number. If plant specimens were collected from the field, describe the collection location, date and sampling procedures.

Novel plant genotypes

Describe the methods by which all novel plant genotypes were produced. This includes those generated by transgenic approaches, gene editing, chemical/radiation-based mutagenesis and hybridization. For transgenic lines, describe the transformation method, the number of independent lines analyzed and the generation upon which experiments were performed. For gene-edited lines, describe the editor used, the endogenous sequence targeted for editing, the targeting guide RNA sequence (if applicable) and how the editor was applied.

Authentication

Describe any authentication procedures for each seed stock used or novel genotype generated. Describe any experiments used to assess the effect of a mutation and, where applicable, how potential secondary effects (e.g. second site T-DNA insertions, mosiacism, off-target gene editing) were examined.

Magnetic resonance imaging

Experimental design

Design type

All studies combined in this manuscript used an fMRI block design.

Design specifications

Studies 1-5 and Study 7 used a mentalizing or related social-cognition task with three conditions: Self-condition, Other-condition, and a non-social Control condition. Study 6a used an inference task with two conditions: attributional and facutla inferences. Study 6b had a 2x2 design with type of inferences (attributional vs factual) and target (social vs nonsocial). The specific task type, the condition specifications, response options, and the number of trials varied across combined studies are delineated in detail in the Method section and summarized in Figure 1.

Behavioral performance measures

No behavioral data is included in this study.

Acquisition

Imaging type(s)

fMRI

Field strength

All of the seven studies used 3T scanners.

Sequence & imaging parameters

All of the seven studies used different data acquisition parameters which are reported in previous publications.
The acquisition parameters in the training dataset are as allows:
The training MRI images were acquired on a 3T Magnetom TIM Trio whole-body scanner (Siemens, Germany) with the product 12-channel head coil. A T1-weighted MPRAGE sequence (TR = 1900ms, TI = 900 ms, TE = 2.27 ms, voxel size 1 x 1 x 1 mm) was used to acquire structural anatomical images. Functional images were obtained using a standard T2-weighted echo-planar imaging sequence (2D-EP, TR = 2100 ms, TE = 30 ms, flip angle 80°, voxel size 3.2 x 3.2 x 3.2 mm) that scanned the whole brain in 36 sequential slices. An automated shimming procedure was included to minimize magnetic field inhomogeneities.

Area of acquisition

Whole brain

Diffusion MRI ☐ Used ☒ Not used

Preprocessing

Preprocessing software

Studies 1,2,3, 5, and 6 used SPM for preprocessing. Study 4 used FSL and Study 7 used fMRIPrep.

Normalization

All studies used comparable co-registration (to structural images) and normalization (to MNI template) procedures. All single-subject contrast images were normalized using L2-norm, and were resampled onto the image space of the training dataset using linear resampling.

Normalization template

MNI template

Noise and artifact removal

Standard noise removal procedures were performed in each studies, including motion parameters in the first-level models as regressors of no interest.

Volume censoring

No volume censoring was used.

Statistical modeling & inference

Model type and settings

During first-level analysis in the training dataset, we included six task (block) regressors that were composed of 3 task conditions by positive and negative valence. The task regressors were convolved with a canonical hemodynamic response function. We also included six additional regressors for motion parameters. A high-pass frequency filter (128s) and autocorrelation corrections (using restricted maximum likelihood and an autoregressive model) were used in model estimation. The validation datasets used different but comparable procedures that are reported in respective publications. Of note, positive and negative valence conditions, in the training dataset, were averaged within each main target condition, ending up with three images (self-, other-, control condition) per participant.

To reduce the possibility that classifiers opportunistically used non-mentalizing related processes (e.g., visual information), we applied a mask in the training dataset that includes key social-cognition regions. This mask was computed as the union of six term-based meta-analytic maps (association and uniformity maps for “mentalizing”, “self-referential”, and “social”, downloaded from NeuroSynth [Yarkoni et al., 2011; https://neurosynth.org] on 06/06/2024).

Single-subject contrast images of the training dataset that is gathered as described above (n=21) were combined with the validation datasets (n=369) for further analysis (total N= 390). All images were normalized at this stage using L2-norm , and resampled onto the same image space as the training datasets.

Effect(s) tested

Training and validation performances (true versus false classification) are tested using ROC plots and binomial tests. Linear mixed effects models were used to test the classification performances across different groups (healthy adults vs individuals with schizophrenia) and to test the associations with age. Repeated-measures ANOVA was used to test the classification performance in Study 7 which used a different social cognition task.

Specify type of analysis: ☐ Whole brain ☐ ROI-based ☒ Both

Anatomical location(s)

The main analyses were computed using a broad social-cognition mask (see above). In addition, we conducted ROI analyses to see the local patterns of self- and other-related mentalizing. To this end, we downloaded a term-based meta-analytic map for ‘Mentalizing’ from NeuroSynth on 14/09/2022 that included 151 studies. We selected clusters that contained more than 200 voxels, resulting in the following ten ROIs: mPFC, bilateral TPJ, bilateral anterior MTG, precuneus/PCC, right SMA, and three clusters in the cerebellum.

Statistic type for inference

(See Eklund et al. 2016)

Prediction across the masked whole brain and ROIs. Voxel-based thresholding of most consistent positive and negative voxels. In addition, general linear (mixed) models i) to compare signatures' performance across different cohorts, and ii) to assess associations with age.

Correction

To illustrate the voxels that contributed to the classifiers most reliably, we applied FDR-correction for multiple comparisons to the bootstrapped images at $q < .05$ with a minimum cluster size of 10 voxels. Following the repeated measures ANOVA in the Study 7, pairwise comparisons between conditions were bonferroni-corrected.

Models & analysis

n/a	Involved in the study
☒	☐ Functional and/or effective connectivity
☒	☐ Graph analysis
☐	☒ Multivariate modeling or predictive analysis

Multivariate modeling and predictive analysis

Using 10-fold cross-validation, we trained three support-vector-machine (SVM) classifiers in Study 1 that discriminate each condition from the other two conditions: The Self-RS was trained to separate the Self-condition from Other and Control conditions, the Other-RS to separate the Other-condition from Self and Control conditions, and the MS was trained to separate both mentalizing conditions (Self and Other) from the Control condition. In addition, we trained one signature to specifically separate the Self from the Other condition (the SvO Signature). The input for SVMs was subject-level contrast images for each of the three task conditions (vs. implicit baseline). Because using a one vs. the rest approach in SVM (e.g., Self vs. Other and Control, see Fig. 1C) may add bias into the model by favoring the majority class, we fitted weighted SVM models with a ridge parameter of .5 to train the Self-RS, the Other-RS, and the MS (but not the SvO-RS). To avoid overfitting, the SVMs were otherwise trained using default parameters (regularization parameter C = 1, linear kernel function, number of folds = 10).

The four mentalizing signatures were applied to the testing datasets by computing the pattern similarity values as the matrix dot product between mentalizing signatures and the subject-level (1st level) contrast images of each study, yielding one scalar value per condition and participant and signature. The predictions followed a binary forced-choice principle using paired observations. The signatures’ classification accuracies were assessed using Receiver Operating Characteristic (ROC) analysis and binomial tests using a two-sided significance threshold of $p < .05$.
