# Supplementary material for: Brain neuromarkers predict self- and other-related mentalizing across adult, clinical, and developmental samples
Source: Nat Commun. 2026 Jun 6;17:7229. doi: 10.1038/s41467-026-73945-w (PMC13396484; doi:10.1038/s41467-026-73945-w)
Supplement: Supplementary file 3 — Transparent Peer Review File [file 41467_2026_73945_MOESM3_ESM.pdf]

nature portfolio

Peer Review File

Brain neuromarkers predict self- and other-related mentalizing across adult, clinical, and developmental samples

Corresponding Author: Dr Leonie Koban

Parts of this Peer Review File have been redacted as indicated to remove third-party material.

This file contains all reviewer reports in order by version, followed by all author rebuttals in order by version.

Version 0:

Reviewer comments:

Reviewer #1

(Remarks to the Author)
I found this an ambitious study that pushes multivariate biomarker approaches into the social cognitive domain. Pooling nine cohorts that span adolescence, typical adulthood, bipolar disorder and schizophrenia is an impressive undertaking and moves MVPA beyond the small, single site studies that dominate the field. The data pipeline is transparent and reproducible, and the analytical path is easy to follow. The developmental finding (more self/other separation with age 11 → 18) and the schizophrenia result (reduced self/other separation) are both theoretically grounded in prior work on self representation and were strong components of the overall analysis.
Although many limitations are already acknowledged by the authors, three issues still stand out for me:
1. Small training set vs. feature space – The core signatures are derived from only 21 participants; even inside the social brain mask this leaves a large sample to voxel mismatch.
2. Prominence of the Mentalizing Signature (MS) – The coarser Self+Other > Control contrast is much easier to classify and risks overshadowing the more theoretically informative—but less robust—Self-RS and Other-RS decoders.
3. Interpretational language – Terms such as “mechanistic neuromarker” and “mentalizing signature” feel stronger than the data warrant, given that the classifiers are trained solely on trait adjective reflection and no perturbation evidence is provided.
Below I expand on each point and suggest feasible additions that, in my view, would bring the paper from very good to compelling.
1. Training set size and cross site robustness
a. A straightforward way to demonstrate that the high accuracies are not buoyed by scanner- or task-specific quirks in the 21-subject discovery set is to add a leave-one-site-out (LOSO) cross-validation analysis. In each fold, the model would be refit on all but one dataset (pooling ~260 participants drawn from several scanners and stimulus variants) and then tested on the completely held-out site. This can quantify how much of the current 75-80 % Self/Other accuracy survives when every discovery-site idiosyncrasy is excluded from training. If accuracy remains comparable, readers gain strong evidence that the signature’s weights capture site-independent neural signal and can be trusted in a new lab. If performance drops, the paper still benefits in that the authors can report a realistic generalization range. It is also not clear why there was no attempt to retrain on the pooled healthy-adult sample (N = 118) to obtain a cleaner, more portable weight map. Implementing this is only a few extra SVM loops and a supplementary table, yet it would materially strengthen the portability claim that underpins the manuscript.
2. Emphasis on the coarse Mentalizing Signature
a. The manuscript highlights the MS—any-person reflection versus heterogeneous low-level controls—as the main success story (98 % accuracy). Because the two classes differ in lexical, semantic and attentional load, that accuracy is not surprising and does not by itself isolate a mentalising mechanism. Meanwhile the Self-RS and Other-RS, which carry the real theoretical weight, receive less attention and understandably show lower performance. I would recommend add two-three sentences explaining the a-priori purpose of the MS in the introduction (e.g., sanity check, transfer to implicit tasks, benchmark ceiling performance). In the Discussion, make clear that MS likely indexes a broad social-reflection state; the self-/other-specific patterns remain the central theoretical tests. Consider balancing Abstract emphasis so that the Self-RS and Other-RS insights are not overshadowed by the universal success of the MS.

3. Wording and scope of the claims
a. The manuscript already notes that the evidence is correlational, yet the repeated phrase “mechanistic neuromarker” still implies causality. I recommend re phrasing to “predictive neural signature” or “candidate biomarker.” Likewise, because every dataset involves trait adjective reflection, it would be fairer to describe the MS classifier as a signature of trait based mentalizing and to state explicitly that generalization to affective, spontaneous or non verbal mentalizing remains to be tested. There was some mention of this in the discussion but the mere use of the term “mentalizing signature” in the rest of the text can be misleading.
The study’s scope, rigor and open workflow make it an important addition to the field. Addressing the training-size issue with a leave-study-out test, re-balancing the emphasis so that the theoretically richer Self-/Other-specific decoders are at the foreground, and tempering causal language would, in my opinion, remove the remaining reservations and position the work for publication in a high-impact venue.

Reviewer #2

(Remarks to the Author)
The authors of this study took a machine learning approach based on support vector machines to analyze fMRI data from multiple cohorts (N = 281), with the aim of identifying robust brain patterns, or “signatures”, associated with “mentalizing” (but - as noted below - the employed datasets rather involve self- versus other-referential processing). To this purpose, they developed and validated classifiers capable of accurately predicting self-referential processing, other-referential processing, and combined self+other referential processing across healthy adults, adolescents, and individuals diagnosed with schizophrenia or bipolar disorder. The combined classifier achieved a predictive accuracy of 98%, while the classifiers focused specifically on self- and other-referential processing reached accuracies of 82% and 77%, respectively, showing both overlapping and distinct neural activation patterns underlying these processes.
Interestingly, the neural segregation between self- and other-referential processing appears to be stronger in healthy adults compared to patients with schizophrenia, and shows improvement with age during adolescence. These results therefore highlight the presence of consistent and distinctive neural markers of self- versus other-referential processing, which may serve as potential bio- or neuro-markers for evaluating social-cognitive functions across developmental trajectories and psychiatric conditions.

In my view, this is a technically sound and well-performed study, with robust analytical methods and findings that hold meaningful implications for future research. However, I see one major issue that should be addressed before the manuscript can be considered for publication in Nature Communications. My concern relates to the putative “exclusivity” of these findings and conclusions to the domain of “mentalizing.” The latter is a broad construct encompassing multiple dimensions, both in terms of underlying cognitive processes and their neural substrates. The datasets/tasks used by the authors for classifier training and validation appears to target a particular facet of this domain, i.e., self- versus other-referential processing. In this respect, it is quite instructive to read the Kelley et al.’s (2002) paper, and to review the subject of the several studies that have quoted it, as most of them address issues that are barely related to “mentalizing”. Broadly speaking, the datasets employed in the present study seem to capture processes related to self-other *judgment*, specifically in the context of attributing internal states. It is therefore not surprising that their classifiers can predict (although to a lesser extent) self- vs. other-related judgments in a social feedback task that is reported to “potentially evoke implicit mentalizing” (but few details are provided on this task). Given the aim of the study, I would have found it more reassuring if the classifiers had failed to predict, or predicted poorly, self- versus other-judgments in a task that does not explicitly involve the self- or other-related attribution of internal states.

Since the authors themselves define mentalizing as “reflecting on one's own and others' internal states”, and given that their datasets appear to involve more generally self- vs. other-referential processing, as a reviewer and reader I would like to see that their classifiers do not accurately distinguish between self- vs. other-judgments unrelated to internal states. Specifically, it would be crucial to demonstrate that the reported classifiers do not accurately distinguish between self- and other-judgments that do not relate to mental states. Although the authors mention control conditions (such as the one depicted in the bottom-right sector of Figure 1), these still appear to involve entities with animacy, which does not fully address this concern. To strengthen the manuscript and its conclusions, I suggest that the authors clarify whether the identified brain signatures are truly specific to mental state attribution or whether they might also predict more generic judgments about the possession of an attribute. Ideally, if an appropriate dataset can be retrieved, the authors could test their classifiers on a task requiring self-other judgments unrelated to mental states, thereby providing a crucial counterproof for their claims. As the lack of this control would decrease the strength of this work, in that case I would recommend that they at least 1) provide negative evidence from "control" ROIs that have been previously associated with a) non-social judgments; b) social non-mentalizing processes; 2) more clearly define the specific facet of "mentalizing" addressed in this study, and 3) explicitly acknowledge this issue as a limitation in their Discussion.

"For the purposes of simplicity and because the pattern of results is in line with the main three signatures, we report the results pertaining to this signature only in the supplementary figure."
--> this is something I hardly understand, given that this comparison embodies the main aim of this study

“The average prediction 261 accuracy in two-choice tests, across all independent validation datasets, was 81.52% for the Self-RS (+/- 6.17% average STE; significant in 10 out of 14 validation tests [7 samples*2comparisons]), 77.25% for the Other-RS (+/- 6.02% average STE; significant in 12 out of 14 tests), ..”
--> please report well-established criteria for assessing the strength of prediction accuracy (and their associated references to published articles)

Please provide more details on the "social feedback" task.

Reviewer #3

(Remarks to the Author)
- What are the noteworthy results?
The SVMs achieved good results on the training set for all 3 classification tasks. The SVMs also achieve good accuracies on the validation sets. It is identified that there is a better self/other separation in healthy adults versus adults with schizophrenia. It is identified that there is a better self/other separation as adolescents age.

- Will the work be of significance to the field and related fields? How does it compare to the established literature? If the work is not original, please provide relevant references.
The work implements a fairly new method to 6 studies in total (1 training + 5 validation). SVMs are not a particularly novel machine learning architecture and the reason for their choice is not well justified either. Thus there are some questions that remain regarding the quality of the work and its significance to brain decoding.
Furthermore, it appears that the authors of the studies where the 5 validation datasets are presents are also authors of the present article. This raises questions regarding their contribution to the present article besides work that has already been published elsewhere.

- Does the work support the conclusions and claims, or is additional evidence needed?
All the conclusions, with the exception of the identified ROIs, are supported by the obtained results. The conclusions regarding the explainability and identification of the most important regions for each class are not wrong or unsupported by the results, but there is a fundamental problem with the method that precludes these conclusions from being valid.

- Are there any flaws in the data analysis, interpretation and conclusions? - Do these prohibit publication or require revision?
Firstly, the manuscript's description of classifier inputs may be misleading. It is my interpretation that the authors feed the SVMs voxel-wise probability estimates derived from a first-level GLM rather than raw BOLD time-series. This critical distinction is buried in a single, vague mention of preprocessing and first-level analysis on page 20 ("SPM8 (Wellcome Department of Imaging Neuroscience, UCL, London, UK) and Matlab® (The MathWorks Inc.) were used for image preprocessing and first-level analysis."), without ever clarifying that classifier features are statistical inputs—not fMRI data. Such obfuscation prevents readers from evaluating whether classification performance reflects genuine neural patterns or merely patterns of the GLM.
Secondly, the claim of a "whole-brain" analysis is entirely circular. Although the text repeatedly emphasizes a whole-brain SVM approach, the authors apply a NeuroSynth-derived mask that restricts voxel selection exclusively to regions associated with mentalizing tasks. By a priori filtering out any voxels unrelated to theory-of-mind paradigms, the study guarantees that the classifier can only "discover" those regions it was forced to consider. As a result, any assertion that the SVM correctly identifies functionally relevant areas is tautological and misleading.
Lastly, the naming of the datasets the model is applied to is confusing. The "training" dataset is used for training and validation of the model's performance, while the "validation" datasets are actually used for testing the model's performance in a deployment setting. It would help if the "validation" datasets were renamed to "testing" or "deployment" datasets.
These flaws fundamentally undermine the manuscript's validity. At minimum, the authors must explicitly state at the outset if the SVM inputs are GLM-based probability maps, not raw fMRI signals, and discuss the interpretive limitations this imposes. They must also either remove the restrictive spatial mask or fully justify its use and reframe all conclusions as conditional on pre-selected mentalizing regions. In its current form, the work does not satisfy the methodological transparency or analytical rigor required for publication.

- Is the methodology sound? Does the work meet the expected standards in your field?
Besides the problems previously stated there are questions regarding the use of SVMs for this analysis. Although SVMs may outperform other trivial machine learning architectures for high dimensional data, they are still a linear architecture. Since brain functioning is characterized by non-linear processes, further justification should be given as to why this architecture was chosen.
Another point that in my opinion seems fragile and requires further explanation is how the weight map from the SVM can be used to extract the importance of each voxel. The authors seem to assume that a higher weight in the SVM means more importance for that voxel. However, these weights are affected by the magnitude of the data points for that voxel, so if the input data is not normalized this assumption may not be verified, invalidating all the conclusions regarding the identification of ROIs.

- Is there enough detail provided in the methods for the work to be reproduced?
Throughout the methods section the authors use terms such as "standard preprocessing pipeline" (line 634) and "default parameters" (line 685). Even if the used values for the parameters are the "standard" or "default" these must still be clearly stated to ensure reproducibility. The authors claim to provide MATLAB code used in the analysis but there's two main problems with this. Firstly, the article must be self-sufficient and readers cannot be expect to go look elsewhere for an acceptable description of the method. Secondly, the URL provided by the authors returns an error, it does not exist. I assume the correct URL should instead be github.com/canlab but even then this is the general GitHub of the laboratory and not of the repository of the code used in this project.
Nowhere in the article is it explained how the Cohen's d values are calculated. Is it using performance metrics of the models? If so for the models with 100% mean accuracy this value should be 0 (since all the models must have 100% accuracy and thus the difference is always 0). So the metrics on which this value are calculated have to be detailed.

Furthermore, in the supplementary materials it seems like high Cohen’s d values (between 2 and 5) are common. Why are these results ignored in the interpretation of the results? Don’t high Cohen’s d values show that the models are inconsistent with each other, i.e. unstable? If this is the case is it safe to extract conclusions regarding the identification of ROIs from unstable models?
It should be cleared if the bootstrapping is run for each fold of the k-fold cross validation. As it stands this is not clear. Furthermore, when the “unthresholded weight maps” are used, are these the results from the original training in the k-fold (before the bootstrapping), the statistical map that results from the bootstrap, or the 5000 weight maps from the bootstrap combined in some way? It is impossible to clearly understand how the models are obtained by following the methods section.
Regarding the k-folds, the authors say there is a 90% for train and 10% for test split. However, since the training set has 21 subjects this would mean there are 2.1 subjects for test. What is 0.1 subjects in this context? Is the split done on a subject or instance level? It must be done on a subject level to insure independence between training and test (to avoid the "double dipping" trap). Moreover 21 subjects means a total of 63 instances for training and testing, assuming the subjects are rounded down (and thus 2 subjects are used for testing) this means there’s only 57 training instances. Is this enough to obtain a generalizable and stable model? Can it be that the reason for high Cohen’s d values is because the training set is not diverse enough in its data to obtain good model stability?
Lastly, and most importantly, it is never clear in any point of the article what the input of the SVMs is. The authors run a first-level (should be "subject-level" not “person-level” in line 705) GLM analysis on the data. The output from the analysis are three contrasts per subject. Each contrast corresponds to one of the three stimuli versus an implicit baseline. What is the implicit baseline? A fixation cross? Random words shown in the same way as the stimuli? This has to be detailed so that the results from the first-level analysis can be understood. Finally, the output from the GLM are statistical parametric maps, which statistics are used? Are these the voxel values that are used as input for the training of the SVM?

In conclusion, there are major oversights in the methods of this work and their description in the article which, in my opinion, precludes its publication.

José Paulo Marques dos Santos

Reviewer #4

(Remarks to the Author)
I co-reviewed this manuscript with one of the reviewers who provided the listed reports. This is part of the Nature Communications initiative to facilitate training in peer review and to provide appropriate recognition for Early Career Researchers who co-review manuscripts.

Version 1:

Reviewer comments:

Reviewer #1

(Remarks to the Author)
Thank you for the thoughtful and comprehensive revision. The new leave one site out analysis using the pooled sample convincingly addresses my main concern about the small discovery set. Reporting AUC and effect size alongside accuracy is very helpful. I also appreciate the rebalancing of the narrative so that the self and other signatures are foregrounded, with the Mentalizing Signature framed as a broad benchmark of social reflection. The revised wording that replaces mechanistic language with predictive or candidate terms, and the explicit clarification that these signatures primarily index trait based mentalizing, bring the claims in line with the evidence. This was an ambitious study and I applaud the authors for the work that was done to address not just my own concerns but also the concerns of the other reviewers. I believe that the paper is much stronger and has a clear and theoretically supported narrative.

With these changes I am satisfied. I have no further requests.

Reviewer #2

(Remarks to the Author)
The authors have put considerable effort into this revision, particularly by adding important analyses (e.g., a leave-one-site-out cross-validation across 5 studies, plus additional generalization tests) and clarifying crucial methodological choices. This work has resulted in an overall improved manuscript.
At the same time, despite these new results, I remain skeptical about the depth of the authors’ interpretation of the findings. As noted in my initial evaluation, my first concern is the interpretation of the results as a selective neural signature of mentalizing. As the authors themselves report in the revised manuscript, their findings provide initial evidence of specificity by showing that the signatures preferentially predict inferences about “hidden” internal states relative to factual judgments, particularly in social contexts. However, above-chance performance also appears in nonsocial attributional conditions. This point should be discussed in the manuscript, and not only in the Response letter. Given the explicit aim of the study and the emphasis on “BRAIN SIGNATURES OF MENTALIZING,” in my view this consideration should be expressed in a brief Limitations section explicitly delimiting claims of specificity. In addition, references to “specificity/selectivity” should be softened across the abstract, results, and discussion.
A similar concern holds for the acknowledgment that the observed “neural signature” pertains primarily to trait-based

mentalizing (i.e., mentalizing about personality traits). While this is now acknowledged in the Introduction and Discussion, I think it should be made explicit in the Abstract with just one more word (if not the title) to more accurately reflect the scope of the authors' inference.

Reviewer #3

(Remarks to the Author)
I appreciate the authors' detailed and thorough replies to the comments and the additional analyses they have performed. Many of the secondary issues I raised have been addressed. However, my principal concern remains unresolved and, in my view, continues to be a critical flaw in the methodological foundation of the work.

Specifically, the authors use subject-level GLM contrast maps as the sole input to the SVM classifiers. While they justify this as standard practice, I remain unconvinced that this approach is appropriate for the claims made in this paper.

While I agree with the authors that preprocessing fMRI data is absolutely necessary, and their rationale for steps such as motion correction, normalization, and filtering is sound, the inclusion of a full first-level GLM analysis within what they describe as preprocessing is problematic. GLM modeling is not a preprocessing step; it is already a statistical analysis that imposes strong assumptions and extracts contrasts of interest. By feeding GLM-derived contrast maps into the SVM, the analysis applies machine learning to data that have already been statistically filtered for their relationship to the experimental conditions. While this may not constitute circularity in the classical sense, it raises concerns about analytical redundancy and makes it difficult to determine what unique information the SVM classification provides beyond the GLM analysis itself.

Moreover, adding a layer of machine learning analysis does not address the underlying limitations of the GLM framework. The intrinsic correlational nature of the analysis remains, and causal inferences about neural mechanisms cannot be drawn from either approach.

Furthermore, in practice, this reduces the classification step to a group-level analysis of GLM-derived maps. Thus, it remains unclear to me what this method provides that could not be achieved through a conventional second-level GLM. If the results from explaining the SVM are comparable to a second-level GLM (the voxels on the first-level maps that are more common for each condition across subjects), I continue to ask myself the question "what is the scientific advancement brought about by this method?", but I still could not find a reassuring answer. The manuscript would be strengthened if the authors could more clearly articulate the unique insights this SVM approach provides beyond conventional second-level GLM analyses. A direct comparison with second-level GLM results would help demonstrate the added value.

Lastly, regarding the usage of masks in the analysis. I still do not see a valid reason for using the "very comprehensive" social cognition mask. In my opinion, the use of a theory-driven mask raises concerns about the validity of any relevant conclusions regarding the importance of each voxel; i.e., circularity persists in the method as voxels are previously screened prior to the application of the statistical tests. Some conclusions may be taken, such as whether a voxel contributes positively or negatively. However, as the authors note, the use of the mask restricts voxels to known ToM areas, thereby precluding the model from capturing potentially relevant activations in less traditional regions, besides inflating the probability of those pertaining to the masks. Therefore, in my opinion, the study now added to the supplementary materials section is much more relevant than the main study of this article, since it uses a whole-brain gray matter mask (the usage of a gray matter mask is biologically plausible, as there should not be BOLD signal in white matter).

For these reasons, despite the otherwise comprehensive revisions, I continue to raise serious concerns about the core of the manuscript maintaining, therefore, the same recommendation.

José Paulo Marques dos Santos

Version 2:

Reviewer comments:

Reviewer #1

(Remarks to the Author)
I have reviewed Reviewer 3's comments, the authors' rebuttal, and the revised manuscript. I believe that the revised submission adequately addresses the core of Reviewer 3's concerns and I do not view the remaining objections as grounds to uphold rejection.

Reviewer 3's main criticism appears to be a broader methodological objection to the use of subject-level first-level GLM contrast maps as inputs to multivariate classifiers. In my view, that is not a fair basis on which to single out this manuscript. Using first-level GLM-derived subject-level maps as classifier inputs is common practice in task-fMRI decoding and brain-signature work, and the authors explain this clearly in their response. They also clarify that first-level GLM estimation and group-level classifier training are distinct, non-circular analytic steps, and they revise the manuscript to make the separation between preprocessing and first-level analysis more explicit. I therefore do not see this as a methodological flaw in the manuscript itself.

I also think the authors have now given an adequate answer to the question of what this approach adds beyond a conventional second-level GLM. The revised paper makes clear that their goal is not simply to identify average voxelwise condition differences, but to build distributed predictive models that can be applied to new data across subjects, datasets, and contexts. That distinction is stated directly in the revised introduction, where they contrast standard brain mapping with multivariate brain signatures that support formally testable population-level prediction. The added leave-one-site-out analyses further strengthen that point by showing that these models retain substantial predictive performance when trained and tested across independent datasets.

On the masking issue, I understand Reviewer 3's general concern that a theory-driven mask constrains discovery. However, I do not think this constitutes fatal circularity here. The mask was defined independently of the current dataset, based on prior empirical literature, and the authors point out that alternative approaches using whole-brain gray-matter masks and other feature-selection strategies yielded similar results. Given that the central theoretical question concerns self- and other-related mentalizing within the broader mentalizing network, I find the masked main analysis defensible, particularly in light of the supplementary whole-brain analyses.

Finally, I think the authors have done a good job addressing the more interpretive concerns that were raised across reviewers. The revised manuscript now explicitly frames the findings as primarily concerning trait-based mentalizing, reduces mechanistic language, and replaces stronger causal phrasing with more appropriate terms such as candidate neuromarkers or predictive brain signatures.

My assessment is that Reviewer 3's remaining objections reflect a more philosophical disagreement with the broader decoding/brain-signature approach than a clear unresolved flaw in this particular manuscript. The authors have responded thoughtfully and substantially, and I believe the revised manuscript is suitable to move forward.

Open Access This Peer Review File is licensed under a Creative Commons Attribution 4.0 International License, which permits use, sharing, adaptation, distribution and reproduction in any medium or format, as long as you give appropriate credit to the original author(s) and the source, provide a link to the Creative Commons license, and indicate if changes were made.
In cases where reviewers are anonymous, credit should be given to 'Anonymous Referee' and the source.
The images or other third party material in this Peer Review File are included in the article's Creative Commons license, unless indicated otherwise in a credit line to the material. If material is not included in the article's Creative Commons license and your intended use is not permitted by statutory regulation or exceeds the permitted use, you will need to obtain permission directly from the copyright holder.
To view a copy of this license, visit https://creativecommons.org/licenses/by/4.0/

Response Letter (NCOMMS-25-21313-T)

Note: Responses to reviewer comments are in blue font.

Reviewer #1 (Remarks to the Author):

I found this an ambitious study that pushes multivariate biomarker approaches into the social cognitive domain. Pooling nine cohorts that span adolescence, typical adulthood, bipolar disorder and schizophrenia is an impressive undertaking and moves MVPA beyond the small, single site studies that dominate the field. The data pipeline is transparent and reproducible, and the analytical path is easy to follow. The developmental finding (more self/other separation with age 11 → 18) and the schizophrenia result (reduced self/other separation) are both theoretically grounded in prior work on self representation and were strong components of the overall analysis.

Although many limitations are already acknowledged by the authors, three issues still stand out for me:

1. Small training set vs. feature space – The core signatures are derived from only 21 participants; even inside the social brain mask this leaves a large sample to voxel mismatch.

2. Prominence of the Mentalizing Signature (MS) – The coarser Self+Other > Control contrast is much easier to classify and risks overshadowing the more theoretically informative—but less robust—Self-RS and Other-RS decoders.

3. Interpretational language – Terms such as "mechanistic neuromarker" and "mentalizing signature" feel stronger than the data warrant, given that the classifiers are trained solely on trait adjective reflection and no perturbation evidence is provided.

Below I expand on each point and suggest feasible additions that, in my view, would bring the paper from very good to compelling.

Response 1.0:

We thank the Reviewer for the encouraging evaluation of our manuscript and constructive comments, which we address in detail below.

1. Training set size and cross site robustness
    a. A straightforward way to demonstrate that the high accuracies are not buoyed by scanner- or task-specific quirks in the 21-subject discovery set is to add a leave-one-site-out (LOSO) cross-validation analysis. In each fold, the model would be refit on all but one dataset (pooling ~260 participants drawn from several scanners and stimulus variants) and then tested on the completely held-out site. This can quantify how much of the current 75-80 % Self/Other accuracy survives when every discovery-site idiosyncrasy is excluded from training. If accuracy remains comparable, readers gain strong evidence that the signature's weights capture site-independent neural signal and can be trusted in a new lab. If performance drops, the paper still benefits in that the authors can report a realistic generalization range. It is also not clear why there was no attempt to retrain on the pooled healthy-adult sample (N = 118) to obtain a cleaner, more portable weight map. Implementing this is only a few extra SVM loops and a supplementary table, yet it would materially strengthen the portability claim that underpins the manuscript.

Response 1.1:

Following this comment, i) we have now added a leave-one-site-out (LOSiO) cross-validation analysis with the pooled dataset (N = 232; n folds = 5), combining Studies 1 to 5 that employed a similar trait evaluation task with three conditions (we used the LOSiO abbreviation to avoid confusion with leave-one-subject-out analysis). These additional results are reported in lines 414-431 and displayed in Figure 9 (see below). In sum, we show that the classification performance remains very good after study-specific variability is accounted for.

Lines 414-431: *"Finally, to ensure that the high accuracies were not an artifact of features unique to the training study (e.g., scanner, sample), we trained new classifiers that combined data across all five training and testing datasets using a leave-one-site-out (LOSiO) cross-validation approach. We combined images from n=232 participants from Studies 1 to 5 that used a trait-evaluation task, pooling data from healthy participants, adolescents, and clinical samples. We used the same parameters to train SVM models corresponding to the four mentalizing signatures.*

Cross-validated prediction accuracies of all four classifiers were very good to excellent (see Fig. 9): 83% averaged accuracy (2AFC) for the Self-RS$_{LOSiO}$, 91% for the Other-RS$_{LOSiO}$, 96% for MS$_{LOSiO}$, and 90% for the SvO-RS$_{LOSiO}$, $p < .001$ (averaged Cohen's d for the Self-RS$_{LOSiO}$ = 1.10, for the Other-RS$_{LOSiO}$ = 1.36, for the MS$_{LOSiO}$ = 0.64, for the SvO-RS$_{LOSiO}$ = 1.19; averaged AUC for the Self-RS$_{LOSiO}$ = 0.90, for the Other-RS$_{LOSiO}$ = 0.95, for the MS$_{LOSiO}$ = 0.93, for the SvO-RS$_{LOSiO}$ = 0.93). Thus, these results show that the high prediction accuracies achieved by the mentalizing signatures were not inflated by study- or site-specific idiosyncrasies but rather reflect consistent, robust multivariate patterns that are achieved using different combinations of the training dataset."

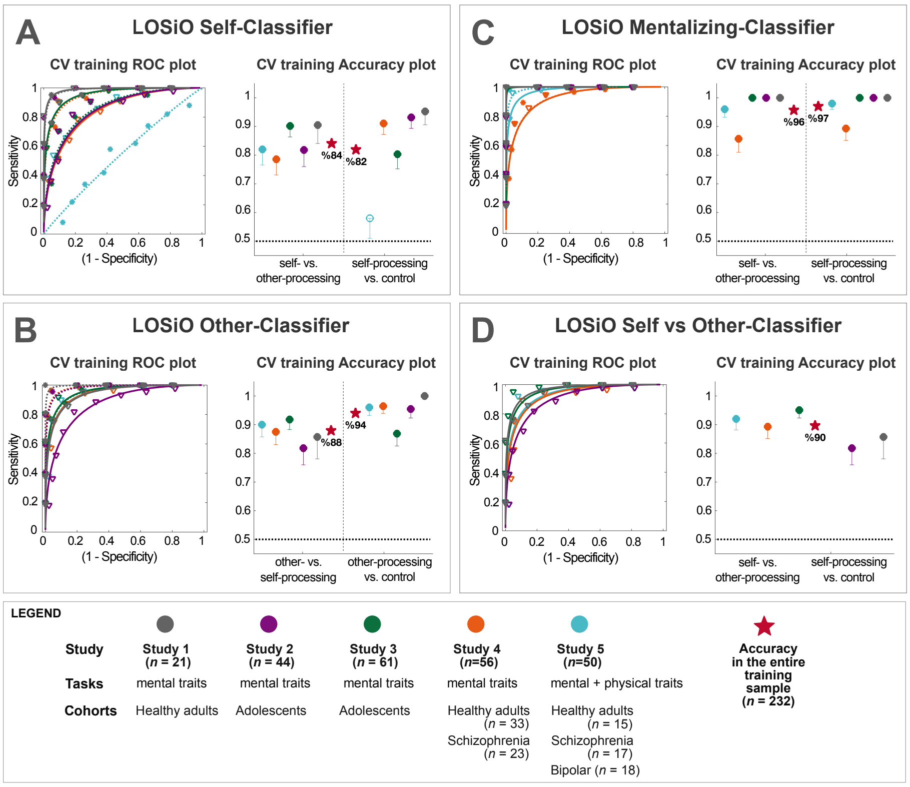

Figure 9 in the revised manuscript.

ii) Another approach the reviewer suggested was to pool the data from the healthy adult samples (n=69, as n=49 of Study 6 used a different task), which produced similar prediction results (see figure below). For conciseness, we decided to report only the LOSiO approach in the revised manuscript, since it leverages a larger and more diverse sample, providing stronger evidence for generalizability.

Together, these additional analyses show that the strong prediction performance and the generalizability of consistent mentalizing brain activity reflect robust multivariate patterns associated with self- and other-related mentalizing. Importantly, the LOSiO classifiers maintained high performance across diverse sites and samples, providing direct evidence that the observed signatures capture site-independent activity patterns.

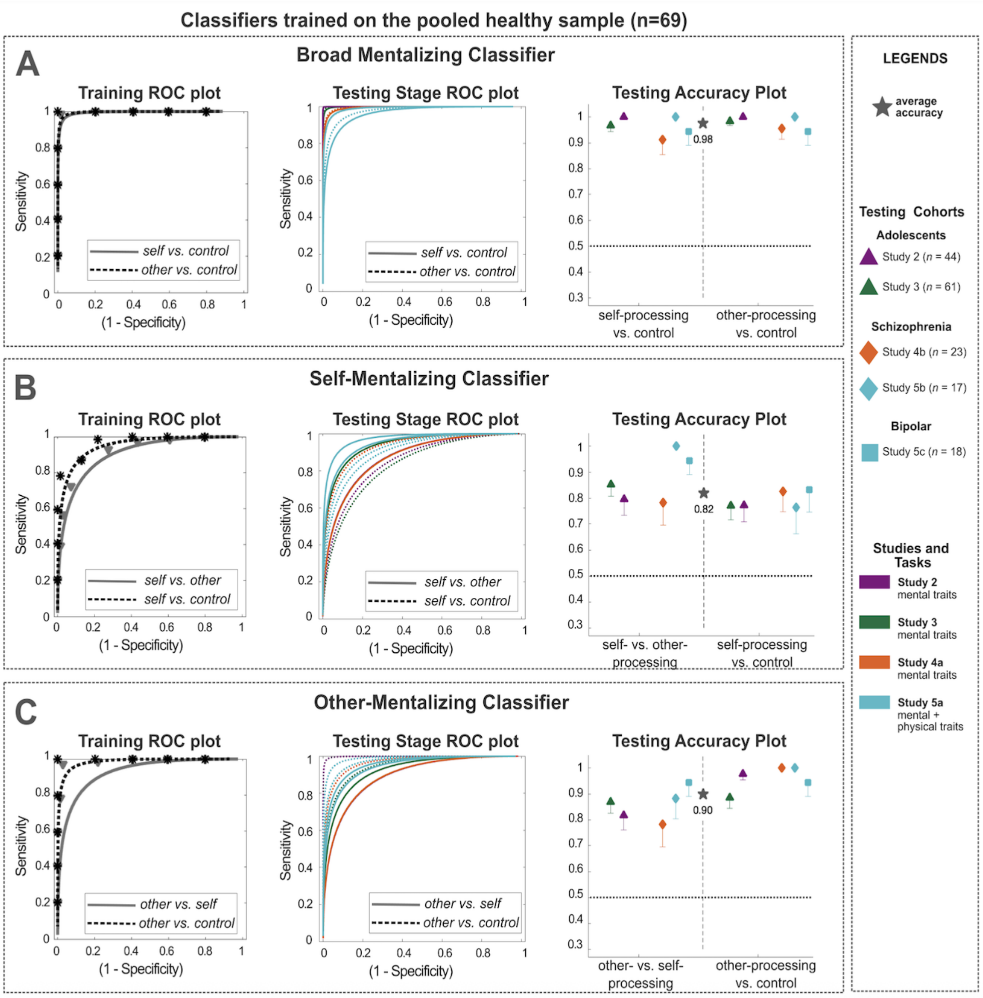

2. Emphasis on the coarse Mentalizing Signature

a. The manuscript highlights the MS—any-person reflection versus heterogeneous low-level controls—as the main success story (98 % accuracy). Because the two classes differ in lexical, semantic and attentional load, that accuracy is not surprising and does not by itself isolate a mentalising mechanism. Meanwhile the Self-RS and Other-RS, which carry the real theoretical weight, receive less attention and understandably show lower performance. I would recommend add two-three sentences explaining the a-priori purpose of the MS in the introduction (e.g., sanity check, transfer to implicit tasks,

benchmark ceiling performance). In the Discussion, make clear that MS likely indexes a broad social-reflection state; the self-/other-specific patterns remain the central theoretical tests. Consider balancing Abstract emphasis so that the Self-RS and Other-RS insights are not overshadowed by the universal success of the MS.

Response 1.2:
We agree that the self-other separation is the harder task and constitutes the core theoretical contribution of this study. As suggested, we have modified the abstract, introduction, and discussion to shift the emphasis in this regard:

In the abstract, we reordered the presentation of signatures (see line 55: *"we apply an interpretable machine learning approach to multiple datasets (total N=281) to train and validate fMRI brain signatures that predict i) mentalizing about the self, ii) mentalizing about another person, and iii) both types of mentalizing."*

In the introduction, we have added a brief explanation of the purpose of the MS (line 150): *"While the overall Mentalizing Signature aimed at developing a broad marker of social reflection that establishes the benchmark ceiling performance and may generalize across a wide range of social processes, the other three signatures (Self-RS, Other-RS, and SvO-RS) allowed us to test whether dissociable and generalizable neural patterns underlie distinct targets of mentalizing."*

In the discussion, we toned down the emphasis we placed on the high performance of the MS and framed this as a broad signature expected to generalize consistently to different social contexts:

Line 436: *"Here, we developed novel brain signatures of self- and other-related mentalizing (Self-RS, Other-RS, and SvO-RS), as well as mentalizing in general (the 'Mentalizing Signature' or MS), combining data ..."*

Line 456: *"The Mentalizing Signature (MS) successfully predicted mentalizing across different contexts and extended to tasks without explicit mentalizing instructions (e.g., spontaneous mentalizing). It also performed very well in separating higher-level inferences about others/hidden states from factual inferences, especially in social contexts, demonstrating its sensitivity to mental state compared to external state attributions."*

3. Wording and scope of the claims

a. The manuscript already notes that the evidence is correlational, yet the repeated phrase "mechanistic neuromarker" still implies causality. I recommend re phrasing to "predictive neural signature" or "candidate biomarker." Likewise, because every dataset involves trait adjective reflection, it would be fairer to describe the MS classifier as a signature of trait based mentalizing and to state explicitly that generalization to affective, spontaneous or non verbal mentalizing remains to be tested. There was some mention of this in the discussion but the mere use of the term "mentalizing signature" in the rest of the text can be misleading. The study's scope, rigor and open workflow make it an important addition to the field. Addressing the training-size issue with a leave-study-out test, re-balancing the emphasis so that the theoretically richer Self-/Other-specific decoders are at the foreground, and tempering causal language would, in my opinion, remove the remaining reservations and position the work for publication in a high-impact venue.

Response 1.3:
We have toned down the claims regarding "mechanistic neuromarker" and replaced them with "candidate" (in the abstract, line 66) and "predictive neuromarkers" (in the discussion, line 574).

We agree that these signatures mainly capture reflective processing on trait-level representations. This is indeed a crucial point we briefly noted earlier in the Introduction (lines 158-166: "*Conceivably, personality traits represent the enduring mental states that can be inferred from information collected by social perceptual systems*[39] *across multiple observations*[12,23]*. Trait representations are used to predict others' future behavior and become the basis of the "mental-self"*[24]*. Moreover, trait evaluations are one of the few mentalizing tasks used in the literature to include a self-condition, as other commonly used tasks (e.g., mental attribution, false-belief, perspective-taking tasks) only permit mentalizing about others. Thus, trait evaluation is an ideally suited task to study a key mentalizing process, namely representing stable psychological states of self and others.").*

We have now edited the discussion to underscore that these signatures are based on trait-based mentalizing:

Line 441: *"First, mentalizing about traits appears to coincide with a specific pattern of brain activity, …"*

Line 556: *"Of note, the mentalizing signatures developed here primarily capture trait-related mentalizing. Here, we provide initial evidence that they extend beyond explicit reflections on trait-level representations. Future studies could test their validity in affective, spontaneous, or non-verbal forms of mentalizing."*

We would like to thank the reviewer again for their positive and thoughtful comments.

Reviewer #2 (Remarks to the Author):

The authors of this study took a machine learning approach based on support vector machines to analyze fMRI data from multiple cohorts (N = 281), with the aim of identifying robust brain patterns, or "signatures", associated with "mentalizing" (but - as noted below - the employed datasets rather involve self- versus other-referential processing). To this purpose, they developed and validated classifiers capable of accurately predicting self-referential processing, other-referential processing, and combined self+other referential processing across healthy adults, adolescents, and individuals diagnosed with schizophrenia or bipolar disorder. The combined classifier achieved a predictive accuracy of 98%, while the classifiers focused specifically on self- and other-referential processing reached accuracies of 82% and 77%, respectively, showing both overlapping and distinct neural activation patterns underlying these processes.

Interestingly, the neural segregation between self- and other-referential processing appears to be stronger in healthy adults compared to patients with schizophrenia, and shows improvement with age during adolescence. These results therefore highlight the presence of consistent and distinctive neural markers of self- versus other-referential processing, which may serve as potential bio- or neuro-markers for evaluating social-cognitive functions across developmental trajectories and psychiatric conditions.

In my view, this is a technically sound and well-performed study, with robust analytical methods and findings that hold meaningful implications for future research. However, I see one major issue that should be addressed before the manuscript can be considered for

publication in Nature Communications. My concern relates to the putative "exclusivity" of these findings and conclusions to the domain of "mentalizing." The latter is a broad construct encompassing multiple dimensions, both in terms of underlying cognitive processes and their neural substrates. The datasets/tasks used by the authors for classifier training and validation appears to target a particular facet of this domain, i.e., self- versus other-referential processing. In this respect, it is quite instructive to read the Kelley et al.'s (2002) paper, and to review the subject of the several studies that have quoted it, as most of them address issues that are barely related to "mentalizing". Broadly speaking, the datasets employed in the present study seem to capture processes related to self-other *judgment*, specifically in the context of attributing internal states. It is therefore not surprising that their classifiers can predict (although to a lesser extent) self- vs. other-related judgments in a social feedback task that is reported to "potentially evoke implicit mentalizing" (but few details are provided on this task). Given the aim of the study, I would have found it more reassuring if the classifiers had failed to predict, or predicted poorly, self- versus other-judgments in a task that does not explicitly involve the self- or other-related attribution of internal states.

Since the authors themselves define mentalizing as "reflecting on one's own and others' internal states", and given that their datasets appear to involve more generally self- vs. other-referential processing, as a reviewer and reader I would like to see that their classifiers do not accurately distinguish between self- vs. other-judgments unrelated to internal states. Specifically, it would be crucial to demonstrate that the reported classifiers do not accurately distinguish between self- and other-judgments that do not relate to mental states. Although the authors mention control conditions (such as the one depicted in the bottom-right sector of Figure 1), these still appear to involve entities with animacy, which does not fully address this concern. To strengthen the manuscript and its conclusions, I suggest that the authors clarify whether the identified brain signatures are truly specific to mental state attribution or whether they might also predict more generic judgments about the possession of an attribute. Ideally, if an appropriate dataset can be retrieved, the authors could test their classifiers on a task requiring self-other judgments unrelated to mental states, thereby providing a crucial counterproof for their claims. As the lack of this control would decrease the strength of this work, in that case I would recommend that they at least 1) provide negative evidence from "control" ROIs that have been previously associated with a) non-social judgments; b) social non-mentalizing processes; 2) more clearly define the specific facet of "mentalizing" addressed in this study, and 3) explicitly acknowledge this issue as a limitation in their Discussion.

Response 2.1:
We thank Reviewer 2 for their positive and constructive comments on our manuscript. The reviewer raises an important point by pointing out that we did not yet have any evidence of exclusivity, or in other words, it was unclear whether the information carried by the mentalizing signatures was specific (or not) to reflecting about internal/mental states. Following their advice, we set out to look for new datasets that would allow us to separate mental state reflection from other types of general reflection about attributes (e.g., more descriptive judgements) and were able to include data from yet another independent study with two cohorts of healthy adults (total n=109 participants):

In these studies, participants were presented with visual stimuli depicting social or nonsocial scenes and asked to answer binary (yes/no) reflection questions. One of the two datasets (n=59; Study 6a) included two experimental conditions: Attributional (e.g., "Is the

person proud of themselves?") vs. factual statements (e.g., "Is the person using both hands?"). The second dataset (n=50, Study 6b) had a 2x2 design with an additional factor of social vs. nonsocial stimuli. While these tasks did not include a self-condition that we would have ideally wished for, they allowed us to test whether mentalizing signatures would be specifically involved in reflecting about hidden (mental) states (attributional statements) vs. observable features (factual statements). Moreover, we could test whether this would be observed especially in social contexts (social targets) or also in nonsocial settings (inanimate targets) that engage these mental processes. We thus tested the predictions of the Other-RS and the MS (overall mentalizing signature) in these two datasets (see new Figure 6 below).

As reported in our revised manuscript (lines 308-335 and new Figure 6), we found that both signatures predict attributional vs. factual inferences in social contexts (i.e., when reflecting about social targets) with excellent accuracies (98% accuracy in 6a and 82% in Study 6b for the Other-RS, and 98% in Study 6a and 100% in Study 6b for the MS in forced-choice tests, *p*<.001). Turning to the nonsocial conditions (available only in Study 6b), interestingly, both signatures still significantly predicted whether participants made attributional vs. factual inferences, but with markedly lower accuracies than in social conditions (66% accuracy for the Other-RS, p=.03, 88% accuracy for the MS, p<.001). Taken together, this shows that mentalizing signatures are indeed implicated in reflective processing such as making inferences about internal/hidden states (attributional processing), for both social and—to a lesser degree—nonsocial targets. It is understandable that attributions in nonsocial contexts also recruit mentalizing circuits, since many underlying suboperations are common to social and nonsocial settings, such as accessing features/attributes of the object, mental stimulation, etc. All in all, we believe that this additional evidence provides initial evidence of specificity, as we illustrate that mentalizing signatures are specifically predictive of reflecting on mental representations of "hidden" inner states rather than cognition guided predominantly by perceptual systems, especially in the social domain when these reflections concern others' inner psychological states.

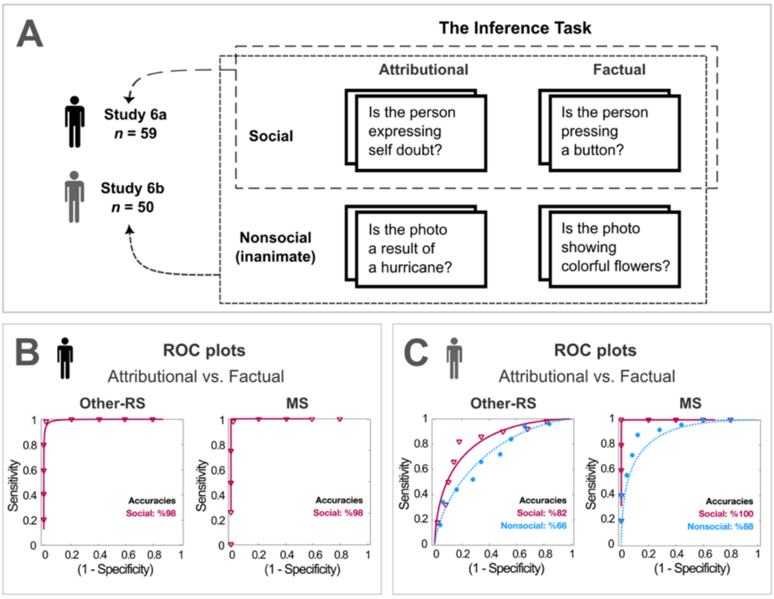

Figure 6 in the revised manuscript.

Another concern raised by Reviewer 2 was whether it was justified to use the term mentalizing for naming the signatures when the training and main validation task was a trait-evaluation task. This is indeed a critical point that we discussed briefly in the Introduction

earlier (see lines 158-166) and are happy to elaborate on again. As widely acknowledged, mentalizing is a construct with multiple dimensions, and our signatures correspond to only a portion of it, which is explicit, cognitive, trait-level mentalizing. Our reasoning for using the term mentalizing to describe these brain signatures was as follows: Personality traits about a person or self are stable mental representations/states that are abstracted from multiple observations. Therefore, trait reflection is mentalizing in the sense that it requires representing inferred psychological states of others (or self). Second, to the best of our knowledge, the trait-evaluation task is one of the few mentalizing tasks used in the literature that includes a self-condition, since other commonly used tasks are based on mentalizing about others (e.g., mental attribution, false-belief, perspective-taking). Therefore, we concluded that our task was suited for our aims and developed the signatures that tap into a specific facet of mentalizing, that is, mentalizing about stable traits of self and others. We agree with the reviewer that this is a critical point in understanding the findings of this study and that it should be stated more clearly throughout the paper.

Thus, in addition to the Introduction (line 158), we have now emphasized this in the Discussion as well:

Line 441: *"First, mentalizing about traits appears to coincide with a specific pattern of brain activity, …"*

Line 556: *"Of note, the mentalizing signatures developed here primarily capture trait-related mentalizing. Here, we provide initial evidence that they extend beyond explicit reflections on trait-level representations. Future studies could test their validity in affective, spontaneous, or non-verbal forms of mentalizing."*

"For the purposes of simplicity and because the pattern of results is in line with the main three signatures, we report the results pertaining to this signature only in the supplementary figure."
--> this is something I hardly understand, given that this comparison embodies the main aim of this study

Response 2.2:
Following this comment, we have now moved the findings regarding the Self-vs-Other signature to the main text (see <u>Fig. 3 and edits in the Results section lines 220, 225, 234, 262, 279, 302</u>). It is now presented alongside the other three signatures, making up the four signatures reported in the main text.

"The average prediction 261 accuracy in two-choice tests, across all independent validation datasets, was 81.52% for the Self-RS (+/- 6.17% average STE; significant in 10 out of 14 validation tests [7 samples*2comparisons]), 77.25% for the Other-RS (+/- 6.02% average STE; significant in 12 out of 14 tests), .."
--> please report well-established criteria for assessing the strength of prediction accuracy (and their associated references to published articles)

Response 2.3:
We previously shared detailed accuracy statistics in the supplementary tables, and following the reviewer's advice, we are now sharing AUC, sensitivity, and specificity in the main text, along with the p-values and the Cohen's d for each classification test (e.g., line 321: *"Attributional (versus factual) inference conditions were predicted with 98% accuracy (two-*

alternative forced-choice) by both the Other-RS and the MS (see Fig. 6B; $p < .001$, AUC = 1.00, sensitivity = 98%; specificity = 98%, Cohen's d = 2.62 for the Other-RS and 3.94 for the MS).").

We have now added more details and a reference regarding well-established criteria for assessing the strength of the prediction accuracy in the Methods section as well (line 810): "*The accuracy rate, area under the curve (AUC), and specificity/sensitivity obtained from these analyses provide the benchmark measures of binary classifications in multivariate modeling (Scheinost et al., 2019). We also report Cohen's d in the text as a more standardized measure to quantify prediction effect sizes across studies and samples. Cohen's d is calculated as the mean of differences between condition values (either pattern expression or distance from the hyperplane) divided by the pooled standard deviation of differences (where difference = input_values[binary_class] - input_values[~binary_class]). Because the three signatures (Self-RS, Other-RS, and MS) were each tested in two comparisons (e.g., Self vs. Other and Self vs. Control), the main text reports the average statistical values across these comparisons, with the complete results provided in the Supplementary Tables. Similarly, when accuracy statistics from a classifier were combined across conditions or studies, we report the weighted average values in the main text and present the full set of statistics in the Supplementary Material.*"

Please provide more details on the "social feedback" task.

Response 2.4:

Thank you for pointing out that the details regarding this task were unclear. We adapted the figure devoted to the results of this task to also include an illustration of the task paradigm (see the figure below). We also edited the text in Results to explain this task more clearly (line 341): *"Participants were led to believe that the task was designed to understand how people rate other people and interpret others' ratings. Therefore, participants first indicated how likable they felt each of 20 individuals (confederates) was, who supposedly rated the participants and their partners in return. To set up a context facilitating spontaneous mentalizing, participants were told that their partners saw the ratings on the screen simultaneously, in a separate room. The task included conditions in which participants received feedback about their own likeability (self-feedback), the likeability of their partner (partner-feedback), as well as a no-feedback control condition. The control condition consisted of trials where no feedback was given, with a prior explanation that such trials signaled that the rater (the individual on the screen) was not shown the participants' picture or did not supply their rating in the time required by the rating task."*

We hope that the figure and the edited text provide more clarity on this paradigm.

REDACTED

We thank Reviewer 2 for the detailed and supportive comments on our manuscript.

Reviewer #3 (Remarks to the Author):

- What are the noteworthy results?
The SVMs achieved good results on the training set for all 3 classification tasks. The SVMs also achieve good accuracies on the validation sets. It is identified that there is a better self/other separation in healthy adults versus adults with schizophrenia. It is identified that there is a better self/other separation as adolescents age.

- Will the work be of significance to the field and related fields? How does it compare to the established literature? If the work is not original, please provide relevant references.
The work implements a fairly new method to 6 studies in total (1 training + 5 validation). SVMs are not a particularly novel machine learning architecture and the reason for their choice is not well justified either. Thus there are some questions that remain regarding the quality of the work and its significance to brain decoding.
Furthermore, it appears that the authors of the studies where the 5 validation datasets are presents are also authors of the present article. This raises questions regarding their contribution to the present article besides work that has already been published elsewhere.

Response 3.1:
Thank you for raising this point. Following this advice, we have now added a more detailed justification for the use of SVMs in our work (line 773): *"SVMs were chosen based on their high performance for binary linear classification problems in high-dimensional and low-sample data (Cox & Savoy, 2003; Misaki et al., 2010; Mourao-Miranda et al., 2005). Linear SVM is a commonly used method in cognitive neuroscience in decoding problems (Oosterwijk et al., 2017; Steardo et al., 2020; Woo et al., 2014), which is computationally efficient and provides interpretable, robust weight maps."*

Regarding authorship, we followed Nature Communications guidelines (https://www.nature.com/ncomms/editorial-policies/authorship). Co-authors of the validation datasets contributed substantially by providing fMRI contrast images, demographic data, and study details, some of which required considerable time and effort. All authors contributed

intellectually by reviewing, editing, and providing conceptual feedback on the manuscript, and all approved its submission.

- Does the work support the conclusions and claims, or is additional evidence needed? All the conclusions, with the exception of the identified ROIs, are supported by the obtained results. The conclusions regarding the explainability and identification of the most important regions for each class are not wrong or unsupported by the results, but there is a fundamental problem with the method that precludes these conclusions from being valid.

Response 3.2:
We appreciate the Reviewer's acknowledgement that all the conclusions were supported by the obtained results. We have clarified below why and how the identification of regions associated with self- and other-related mentalizing is valid and not problematic. With respect, we have a different opinion on whether these arguments challenge the validity of our conclusions. We hope that our responses below provide more clarity.

- Are there any flaws in the data analysis, interpretation and conclusions? - Do these prohibit publication or require revision?
Firstly, the manuscript's description of classifier inputs may be misleading. It is my interpretation that the authors feed the SVMs voxel-wise probability estimates derived from a first-level GLM rather than raw BOLD time-series. This critical distinction is buried in a single, vague mention of preprocessing and first-level analysis on page 20 ("SPM8 (Wellcome Department of Imaging Neuroscience, UCL, London, UK) and Matlab® (The MathWorks Inc.) were used for image preprocessing and first-level analysis."), without ever clarifying that classifier features are statistical inputs—not fMRI data. Such obfuscation prevents readers from evaluating whether classification performance reflects genuine neural patterns or merely patterns of the GLM.

Response 3.3:
As suggested, we have now explained our approach more clearly in the revised manuscript (see Figure 1 below, line 758: *"The input for SVMs was subject-level contrast images for each of the three task conditions (versus implicit baseline)"* and line 711: *"As it is the standard practice in the development of brain signatures (Wager et al, 2013; Kragel et al., 2018, Koban et al, 2023), we used statistical maps from first-level analysis (e.g., single-subject contrast images) as input for training and testing the classifiers.").*

It is not only standard but non-negotiable in fMRI research to preprocess BOLD data, which can be strongly affected by movement artefacts and other sources of noise. We would like to point out that "raw BOLD time-series" is de facto already a reconstructed image and a highly transformed signal from what was originally a k-space signal. Further, the fMRI BOLD signal reflects the balance of deoxyhemoglobin versus oxyhemoglobin, which have different paramagnetic properties. Therefore, fMRI cannot directly measure neural patterns, but it can measure brain activity indirectly and relatively. It is therefore standard for the current approach of brain signature development (e.g., Wager, et al., 2013 *NEJM*; Woo et al., 2014 *Nat Comms*; Krishnan et al. 2016 *eLife*; Kragel et al. 2018, *Neuron*; Koban et al., 2023 *Nat Neurosci*, and others) and other types of fMRI-based decoding (Haynes, 2015, *Neuron;* Scheinost et al. 2019, *NeuroImage*) to work with contrast images, which are the most rigorous estimate of

‘brain activation’ to an experimental condition that we can obtain, precisely because the GLM statistically isolates the portion of the BOLD signal driven by the condition of interest (e.g., thinking about the self) and what is driven by other sources (e.g., signal drifts, movement artifacts, button presses).

Therefore, in our study, the SVM classifiers were trained on voxel-wise beta estimates from first-level GLM analyses (i.e., contrast images). These beta maps represent the modeled response amplitude for each experimental condition, thus summarizing task-related neural activation patterns in a way that reduces noise and accounts for nuisance regressors. Our revised text and Fig. 1 (below) now clarifiy this (e.g., lines 185, 711, 758, 807) to make it unambiguously clear for the readers.

A

	Sample		Task Features					Scanner
	Cohort characteristics	*n*	*Task type*	*Other-mentalizing condition*	*Control condition*	*Response type*	*Number of trials*	
Training Dataset — Study 1	Healthy adults (M_{age} =24)	n = 21	Trait evaluation task (TET) - adj.	Unfamiliar confederate	Count the syllables	Likert scale (1-4)	30 blocks * 5 trials	3T Siemens Trio
Testing Datasets — Study 2	Adolescents (M_{age} = 16)	n = 44	TET - trait adjectives	Best friend	Count the syllables	Likert scale (1-4)	30 blocks * 10 trials	3T Siemens Trio
Study 3	Adolescents (M_{age} = 13)	n = 61	TET - trait adjectives	Similar & dissimilar classmate	Letter present or not	Binary choice	32 blocks * 5 trials	3T Philips Ingenia CX
Study 4a	Healthy adults (M_{age} = 42)	n = 33	TET - trait statements	Acquaintance	General knowledge	Binary choice	6 blocks * 9 trials	3T Philips Achieva
Study 4b	Schizophrenia patients (M_{age} = 37)	n = 23	TET - trait statements	Acquaintance	General knowledge	Binary choice	6 blocks * 9 trials	3T Philips Achieva
Study 5a	Healthy adults (M_{age} = 33)	n = 15	TET - trait & physical statements	Relative or close friend	General knowledge	Likert scale (1-4)	36 blocks * 5 trials	3T Philips Intera
Study 5b	Schizophrenia patients (M_{age} = 36)	n = 17	TET - trait & physical statements	Relative or close friend	General knowledge	Likert scale (1-4)	36 blocks * 5 trials	3T Philips Intera
Study 5c	Bipolar patients (M_{age} = 40)	n = 18	TET - trait & physical statements	Relative or close friend	General knowledge	Likert scale (1-4)	36 blocks * 5 trials	3T Philips Intera
Extension Datasets — Study 6a	Healthy adults (M_{age} = 28)	n = 59	Inference task	Strangers	Factual inference	Binary choice	8 blocks * 8 trials	3T Siemens Trio
Study 6b	Healthy adults (M_{age} = 34)	n = 50	Inference task	Strangers	Factual inference	Binary choice	24 blocks * 9 trials	3T Siemens Prisma
Study 7	Healthy adults (M_{age} = 23)	n = 49	Social feedback task	Romantic partner	No feedback	Passive viewing	45 blocks * 5 trials	3T Siemens Skyra

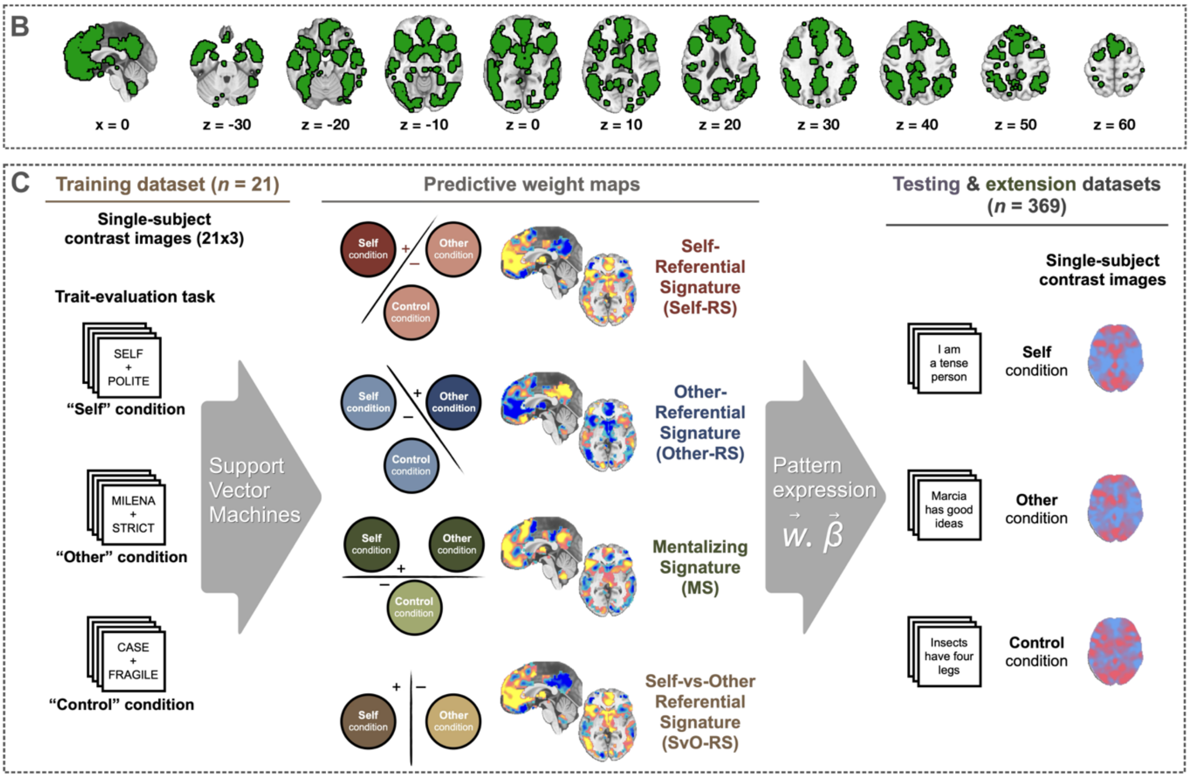

Secondly, the claim of a “whole-brain” analysis is entirely circular. Although the text repeatedly emphasizes a whole-brain SVM approach, the authors apply a NeuroSynth-derived mask that

restricts voxel selection exclusively to regions associated with mentalizing tasks. By a priori filtering out any voxels unrelated to theory-of-mind paradigms, the study guarantees that the classifier can only "discover" those regions it was forced to consider. As a result, any assertion that the SVM correctly identifies functionally relevant areas is tautological and misleading.

Response 3.4:
We have now corrected the usage of "whole-brain" in the text, which indeed added confusion to our approach. It was important to underline that the classifiers use information from all available voxels, for which we now use the word "distributed" (see line 179: "distributed *brain signatures of mentalizing").*

Regarding the use of a mask, it is crucial to highlight that the feature selection was not performed on the datasets included in the study; therefore, it was completely independent from the rigorous cross-validations, and out-of-sample predictions. Since the main goal of our work was to identify self- and other-referential brain activity, a (very comprehensive) mask was chosen to reduce the influence of voxels unrelated to social cognition that the classifier could opportunistically pick up on. However, we agree that it can be informative to repeat our analyses using a whole-brain gray-matter mask, which we have now added to the Supplementary Material (also see below) showing that the key findings remain unchanged: The predictive performance of whole-brain classifiers are highly comparable to our main masked classifiers and the significant weights overlap to a great extent in two versions. This suggests that the use of the mask did not substantially alter the results, and that our brain signatures are robust patterns that replicate across different analytic choices. While it is true that, by masking, we exclude the possibility of including any areas outside of the mask to make inferences about involved areas, we would like to outline that we separately conducted literature-driven ROI analysis for inferences about brain regions (see Fig. 8 in the manuscript).

In conclusion, the additional results from the whole-brain classifiers show that our results are not due to a circular analytic strategy. Since i) we combine information obtained from the training brain-wide classifiers (Fig. 2) and ROI classifiers (Fig. 8) for inferences about brain regions and ii) additional whole-brain classifiers also indicate similar regions, together, these analyses provide a robust evidential basis for our regional inferences. We acknowledge, however, that the use of mask should be considered in understanding our results, so we edited the text in Methods as follows (see line 767: *"While the use of a mask was intended to minimize involvement of the confounding voxels that are not directly related to social cognition, this might raise the question of how more extensive, whole-brain classifiers might perform. As shown in Supplementary Fig. 1, applying a whole-brain gray matter mask yielded highly similar results. ").*

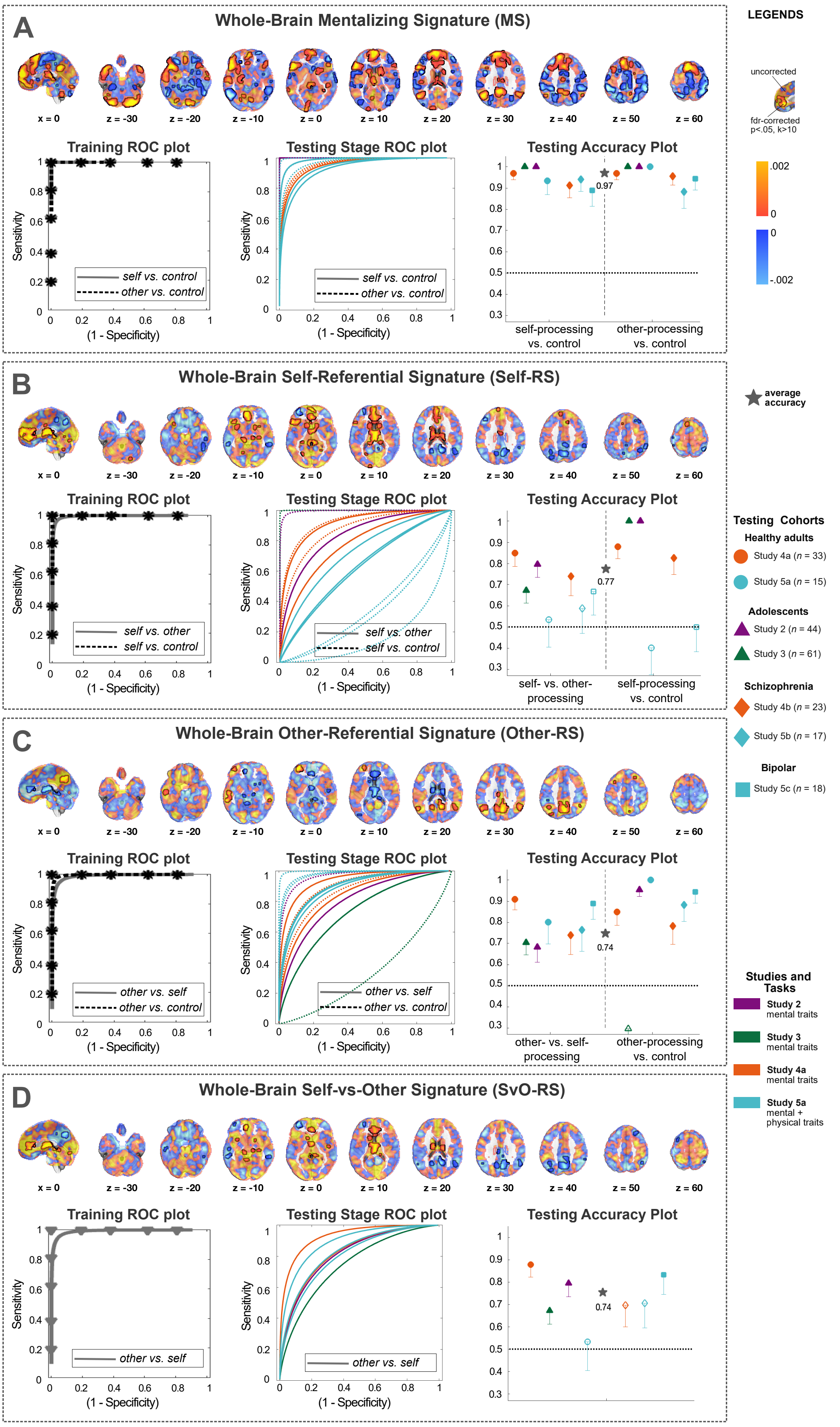
A
Whole-Brain Mentalizing Signature (MS)
B
Whole-Brain Self-Referential Signature (Self-RS)
C
Whole-Brain Other-Referential Signature (Other-RS)
D
Whole-Brain Self-vs-Other Signature (SvO-RS)
x = 0
z = -30
z = -20
z = -10
z = 0
z = 10
z = 20
z = 30
z = 40
z = 50
z = 60
Training ROC plot
Testing Stage ROC plot
Testing Accuracy Plot
Sensitivity
(1 - Specificity)
self vs. control
other vs. control
self vs. other
other vs. self
0.97
0.77
0.74
0.74
self-processing vs. control
other-processing vs. control
self- vs. other-processing
other- vs. self-processing
LEGENDS
uncorrected
fdr-corrected p<.05, k>10
.002
0
0
-.002
average accuracy
Testing Cohorts
Healthy adults
Study 4a (n = 33)
Study 5a (n = 15)
Adolescents
Study 2 (n = 44)
Study 3 (n = 61)
Schizophrenia
Study 4b (n = 23)
Study 5b (n = 17)
Bipolar
Study 5c (n = 18)
Studies and Tasks
Study 2 mental traits
Study 3 mental traits
Study 4a mental traits
Study 5a mental + physical traits

Lastly, the naming of the datasets the model is applied to is confusing. The “training” dataset is used for training and validation of the model’s performance, while the “validation” datasets are actually used for testing the model’s performance in a deployment setting. It would help if the “validation” datasets were renamed to “testing” or “deployment” datasets.

Response 3.5:
Following this comment, we have renamed the datasets as “cross-validated training”, “testing datasets”, and “extension datasets” (please see the figure in Response 3.3). The testing datasets refer to datasets that used the same task as the training dataset, and extension datasets refer to datasets that used distinct tasks where mentalizing is not explicitly instructed, which are used to test whether classifiers “extend” to these contexts.

These flaws fundamentally undermine the manuscript’s validity. At minimum, the authors must explicitly state at the outset if the SVM inputs are GLM-based probability maps, not raw fMRI signals, and discuss the interpretive limitations this imposes. They must also either remove the restrictive spatial mask or fully justify its use and reframe all conclusions as conditional on pre-selected mentalizing regions. In its current form, the work does not satisfy the methodological transparency or analytical rigor required for publication.

Response 3.6:
As noted above (Response 3.3), the use of GLM outputs is not a limitation, but a standard procedure in the fMRI-based decoding studies. Following the reviewer’s suggestion, we nonetheless explicitly state now that the SVM inputs are GLM outputs (line 710): *“As it is the standard practice in the development of brain signatures (Wager et al, 2013; Kragel et al., 2018, Koban et al, 2023), we used statistical maps from first-level analysis (e.g., single-subject contrast images) as input for training and testing the classifiers.”* and in line 757: *“The input for SVMs was subject-level contrast images for each of the three task conditions (vs. implicit baseline).“*

As outlined in Response 3.4, our additional analyses with a whole-brain mask show that our results were not a product of the mask, as they yielded comparable predictive performance and largely overlapping voxel weights. Taken together, we believe these clarifications and additional analyses address the reviewer’s concerns and further support the rigor of our approach and the validity of our conclusions.

- Is the methodology sound? Does the work meet the expected standards in your field? Besides the problems previously stated there are questions regarding the use of SVMs for this analysis. Although SVMs may outperform other trivial machine learning architectures for high dimensional data, they are still a linear architecture. Since brain functioning is characterized by non-linear processes, further justification should be given as to why this architecture was chosen.

Response 3.7:
As described above (Response 3.1), we have now extended our explanation of why we used SVMs for our analyses (see line 772). Linear classifiers have several advantages that make them well-suited for our aims. First, in practice, linear algorithms often outperform non-linear algorithms and features, potentially because they are less likely to overfit (see Cox & Savoy,

2003, *NeuroImage*; Misaki et al., 2010, *NeuroImage)*. While brain processes themselves may be nonlinear, this does not necessarily translate directly to the BOLD signal measured with fMRI. Second, our aim was to develop brain signatures that are interpretable and generalizable across diverse contexts, a goal for which linear algorithms are particularly appropriate (Kragel et al., 2018, Neuron). Third, linear models are more transparent and interpretable than nonlinear models, which often face the 'black-box' problem and can rely on spurious features. Therefore, considering the aim of this study, that is, to derive conceptually meaningful and usable brain signatures of self- and other-referential mentalizing, we consider SVMs the most suitable choice for the current study.

Another point that in my opinion seems fragile and requires further explanation is how the weight map from the SVM can be used to extract the importance of each voxel. The authors seem to assume that a higher weight in the SVM means more importance for that voxel. However, these weights are affected by the magnitude of the data points for that voxel, so if the input data is not normalized this assumption may not be verified, invalidating all the conclusions regarding the identification of ROIs.

Response 3.8:

We are happy to clarify this point here. As stated in the manuscript, the input data have been normalized using L2-norm (see line 197: *"Contrast images were computed for each condition (versus implicit baseline) and rescaled using L2-norm to standardize the scale of beta (regression coefficient) weights across participants, studies, and scanners."* & line 739: *"Voxel weights were normalized within each image using L2-norm, and contrast images were resampled onto the same image space as the training dataset using linear resampling."*).

Therefore, the thresholded weight maps are useful in illustrating voxels with the strongest positive and negative contributions to the predictions. We agree that this might not necessarily mean importance per se. This is why we decided to compute ROI classifiers (see Fig. 8 in the revised manuscript) to test whether local patterns have consistent and robust weights that generalize to independent datasets. Only by combining these two sources of information, we make inferences about the involvement of a particular region during a specific condition.

An alternative approach would be to compute weight maps using recursive feature elimination (RFE). RFE is a method that can be used for feature selection in the SVM context, which aims to identify a subset and combination of features/voxels that have the best predictive power. Below, we present the RFE-derived weight maps (final n = 2000 features, cluster size k > 20) for the four mentalizing classifiers, each corresponding to the models with the highest predictive performance in their respective cross-validated training. The resulting patterns of positive and negative weights are largely consistent with those identified in the thresholded weight maps of the mentalizing signatures. Of note, an RFE model eventually ends up using final *n* features (2000 in this case), which is less ideal for the brain signatures approach (see Kragel et al., 2018) that we undertook, which favors brain-wide, global models over limited, specific models.

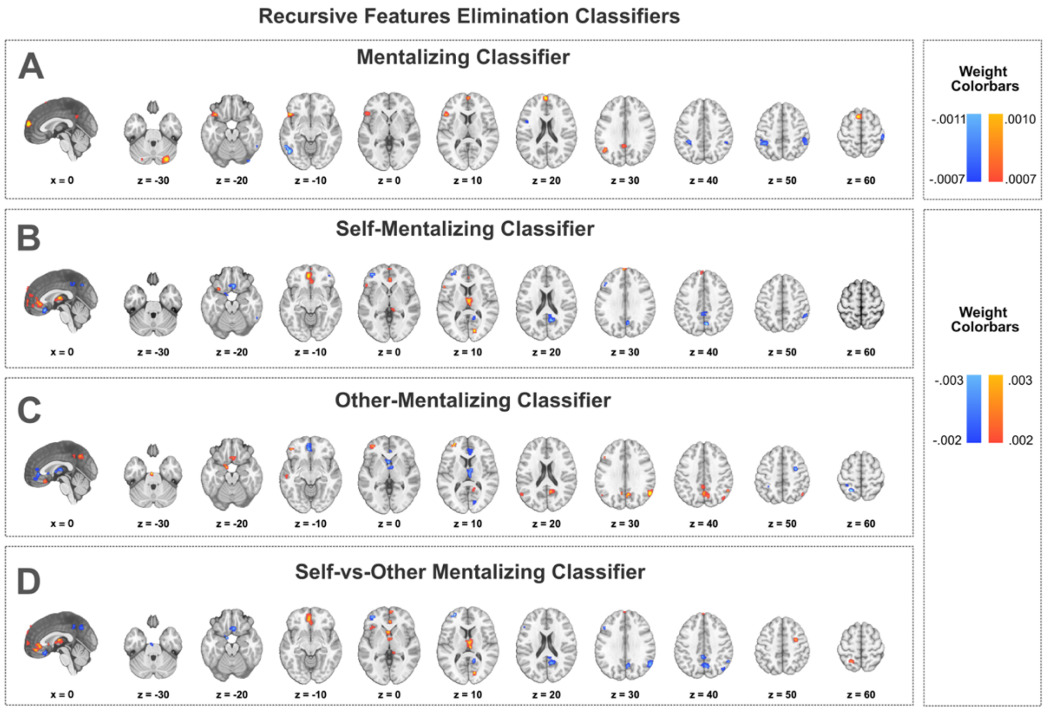

New Supplementary Figure 2

- Is there enough detail provided in the methods for the work to be reproduced? Throughout the methods section the authors use terms such as “standard preprocessing pipeline” (line 634) and “default parameters” (line 685). Even if the used values for the parameters are the “standard” or “default” these must still be clearly stated to ensure reproducibility.

Response 3.9:

As suggested, we expanded the description of the default or standard parameters in the revised Methods:

Line 781: *“Because using a one vs. the rest approach in SVM (e.g., Self vs. Other and Control, see Fig. 1C) may add bias into the model by favoring the majority class, we fitted weighted SVM models with a ridge parameter of .5 to train the Self-RS, the Other-RS, and the MS (but not the SvO-RS). To avoid overfitting, the SVMs were otherwise trained using default parameters (regularization parameter C = 1, linear kernel function, number of folds = 10).”*

Regarding the fMRI preprocessing and first-level analysis steps, we report the following information in the Method section:

Line 703: *“The training MRI images were acquired on a 3T Magnetom TIM Trio whole-body scanner (Siemens, Germany) with the product 12-channel head coil. A T1-weighted MPRAGE sequence (TR = 1900ms, TI = 900 ms, TE = 2.27 ms, voxel size 1 x 1 x 1 mm) was used to acquire structural anatomical images. Functional images were obtained using a standard T2-weighted echo-planar imaging sequence (2D-EP, TR = 2100 ms, TE = 30 ms, flip angle 80°, voxel size 3.2 x 3.2 x 3.2 mm) that scanned the whole brain in 36 sequential slices. An automated shimming procedure was included to minimize magnetic field inhomogeneities.*

As it is the standard practice in the development of brain signatures (Wager et al, 2013; Kragel et al., 2018; Koban et al, 2023), we used statistical maps from first-level analysis (e.g., single-subject contrast images) as input for training and testing the classifiers. SPM8

(Wellcome Department of Imaging Neuroscience, UCL, London, UK) and Matlab® (The MathWorks Inc.) were used for image preprocessing and first-level analysis. A standard preprocessing pipeline was performed, which included spatial realignment and reslicing of the functional images, coregistration, unified segmentation and normalization to the Montreal Neurological Institute (MNI) echo planar imaging template (voxel size: 2 mm^3), and finally spatial smoothing using an 8 mm Full Width at Half Maximum (FWHM) Gaussian kernel.

During first-level analysis, we included six task (block) regressors that were composed of 3 task conditions by positive and negative valence. The task regressors were convolved with a canonical hemodynamic response function. We also included six additional regressors of no interest to control for motion. A high-pass frequency filter (128s) and autocorrelation corrections (using restricted maximum likelihood and an autoregressive model) were used in model estimation."

We would be glad to provide additional parameters if there are still any missing.

The authors claim to provide MATLAB code used in the analysis but there's two main problems with this. Firstly, the article must be self-sufficient and readers cannot be expect to go look elsewhere for an acceptable description of the method. Secondly, the URL provided by the authors returns an error, it does not exist. I assume the correct URL should instead be github.com/canlab but even then this is the general GitHub of the laboratory and not of the repository of the code used in this project.

Response 3.10:
We apologize for the incorrect URL and have now added a more project-specific code repository containing all scripts necessary to reproduce the analyses, as also requested by the journal: https://github.com/ldmk/2025_MentalizingSignatures

We fully agree that the article itself must be self-sufficient to be able to be replicated. Thus, the shared scripts are intended to accompany a clear Method section. To ensure this, we have now added further details in the revised manuscript, as suggested by the reviewer.

Nowhere in the article is it explained how the Cohen's d values are calculated. Is it using performance metrics of the models? If so for the models with 100% mean accuracy this value should be 0 (since all the models must have 100% accuracy and thus the difference is always 0). So the metrics on which this value are calculated have to be detailed. Furthermore, in the supplementary materials it seems like high Cohen's d values (between 2 and 5) are common. Why are these results ignored in the interpretation of the results? Don't high Cohen's d values show that the models are inconsistent with each other, i.e. unstable? If this is the case is it safe to extract conclusions regarding the identification of ROIs from unstable models?

Response 3.11:
Cohen's d is a standard effect size measure and does not index model instability; rather, it reflects the strength of the classification. For example, in a t-test, the larger the difference between two conditions, the higher the Cohen's d. Conventionally, values below 0.3 are considered small, around 0.5 moderate, and above 0.8 large effects. In our analyses, Cohen's d reflects how well the classifiers separate two different conditions (and not, as suggested by the reviewer, the difference between models). We calculated Cohen's d as the difference in classifier response for the two conditions to be separated (d = mean of differences / pooled

standard deviation of differences). Therefore, the high Cohen's d values (often above 2) show the strong effect sizes and, therefore, the practical usefulness of the classifiers in correctly classifying different conditions.

We have added a brief explanation of Cohen's d in the revised Methods section (see line 814): *"Cohen's d is calculated as the mean of differences between condition values (either pattern expression or distance from the hyperplane) divided by the pooled standard deviation of differences (where difference = input_values[binary_class] - input_values[~binary_class])."*

It should be cleared if the bootstrapping is run for each fold of the k-fold cross validation. As it stands this is not clear. Furthermore, when the "unthresholded weight maps" are used, are these the results from the original training in the k-fold (before the bootstrapping), the statistical map that results from the bootstrap, or the 5000 weight maps from the bootstrap combined in some way? It is impossible to clearly understand how the models are obtained by following the methods section.

Response 3.12:

Thank you for pointing this out. Bootstrapping was applied after the cross-validation stage, using the full training dataset, and serves to obtain voxel-wise *p*-values for the model weights. In the first step, the SVM model is trained once with cross-validation. A final model output (weight map) is created at this stage using data from all samples, which constitute the unthresholded weight maps. In the next step, this weight map is resampled 5000 times during bootstrapping to produce voxel-wise distributions of estimates, allowing the calculation of p-values. Thus, the unthresholded statistical weight maps resulting from the cross-validation procedure are the mentalizing signatures that are used for prediction in independent datasets, whereas thresholded maps are used for anatomical inference and visualization. We edited the text to clarify our Method section (see line 794: *"We used bootstrapping to train SVM classifiers, producing stable model estimates and voxel-wise p-values. In the first step, cross-validated training generated prediction accuracies and a final model output (i.e., weight map) that is trained on all samples. In the next step, the bootstrapping procedure resampled the training data 5000 times with replacement, yielding statistical maps with two-tailed, uncorrected p-values. These maps were then thresholded using FDR-correction at q < .05 with a minimum cluster size of 10 voxels. Note that corrected maps were only used for visualization and inference. The unthresholded weight maps constitute the mentalizing signatures and, therefore, are used for analysis."*).

Regarding the k-folds, the authors say there is a 90% for train and 10% for test split. However, since the training set has 21 subjects this would mean there are 2.1 subjects for test. What is 0.1 subjects in this context? Is the split done on a subject or instance level? It must be done on a subject level to insure independence between training and test (to avoid the "double dipping" trap). Moreover 21 subjects means a total of 63 instances for training and testing, assuming the subjects are rounded down (and thus 2 subjects are used for testing) this means there's only 57 training instances. Is this enough to obtain a generalizable and stable model? Can it be that the reason for high Cohen's d values is because the training set is not diverse enough in its data to obtain good model stability?

Response 3.13:

During cross-validation, all data (contrasts) from a given subject were always held out together. Since 21 subjects were used for training, 9 folds included the data of 2 subjects, and 1 fold included the data of 3 subjects, so that all subjects' data were used for training and testing. We have now clarified this in the Method section (see line 758): *"The cross-validation folds were organized to ensure that the three contrast images from each participant were kept together within a single fold (nine folds with two participants and one fold with three participants)"* and the Results section (line 215): *"The folds were defined at the subject level, so that all contrast images from a given subject were assigned to the same fold."*

Regarding Cohen's d, as outlined in Response 3.11, these values index the effect size of classification (i.e., separation between conditions), not instability of the models. High values, therefore, indicate a robust model that generalizes to completely independent datasets. The concern about a generalizable model was also raised by Reviewer 1 (please see Response 1.1), following which we conducted additional leave-one-site-out (LOSiO) cross-validation analysis. This provided supporting evidence that the predictive performance of the mentalizing signatures is not driven by task-specific idiosyncrasies, but instead reflects consistent, brain-wide patterns that replicate across different permutations of the dataset.

Lastly, and most importantly, it is never clear in any point of the article what the input of the SVMs is. The authors run a first-level (should be "subject-level" not "person-level" in line 705) GLM analysis on the data. The output from the analysis are three contrasts per subject. Each contrast corresponds to one of the three stimuli versus an implicit baseline. What is the implicit baseline? A fixation cross? Random words shown in the same way as the stimuli? This has to be detailed so that the results from the first-level analysis can be understood. Finally, the output from the GLM are statistical parametric maps, which statistics are used? Are these the voxel values that are used as input for the training of the SVM?

Response 3.14:

We apologize if the input of the SVM was not described clearly enough. As is standard in fMRI analysis, our first step is to run a subject-level GLM to statistically identify brain activation for each experimental condition (Self, Other, Control) versus an implicit baseline. "Implicit baseline" is indeed a jargon term in the fMRI field that refers to activity that is not modeled by the GLM, such as periods between task blocks (e.g., when participants viewed only the fixation cross). Indeed, the output of the GLM is so-called beta maps, which describe how much each voxel's BOLD signal is positively or negatively influenced by each regressor (i.e., to each experimental condition). These maps are the closest we can get to measuring 'brain activation' using fMRI. Therefore, these subject-level contrast maps constituted the input for the training of SVMs.

In the revised manuscript, we adapted the term "subject-level" and clarified our approach in the Method section, see line 757: *"The input for SVMs was subject-level contrast images for each of the three task conditions (versus implicit baseline). The cross-validation folds were organized to ensure that the three contrast images from each participant were kept together within a single fold (nine folds with two participants and one fold with three participants)."* and line 710: *"As it is the standard practice in the development of brain signatures (Wager et al, 2013; Kragel et al., 2018, Koban et al, 2023) to work with contrast images, we used statistical maps from first-level analysis (e.g., single-subject contrast images) as input for training and testing the classifiers."*

In conclusion, there are major oversights in the methods of this work and their description in the article which, in my opinion, precludes its publication.

José Paulo Marques dos Santos

Response 3.15:
We thank the reviewer for his detailed reading of our manuscript and for many constructive comments that helped us helped us further clarify the description of our methods and results.

Reviewer #4 (Remarks to the Author):

I co-reviewed this manuscript with one of the reviewers who provided the listed reports. This is part of the Nature Communications initiative to facilitate training in peer review and to provide appropriate recognition for Early Career Researchers who co-review manuscripts.

Response 4.1:
We thank all four reviewers for their time, their careful reading of our manuscript, and their thoughtful and constructive comments.

Response Letter

NCOMMS-25-21313A

Reviewer #1 (Remarks to the Author):

Thank you for the thoughtful and comprehensive revision. The new leave one site out analysis using the pooled sample convincingly addresses my main concern about the small discovery set. Reporting AUC and effect size alongside accuracy is very helpful. I also appreciate the rebalancing of the narrative so that the self and other signatures are foregrounded, with the Mentalizing Signature framed as a broad benchmark of social reflection. The revised wording that replaces mechanistic language with predictive or candidate terms, and the explicit clarification that these signatures primarily index trait based mentalizing, bring the claims in line with the evidence. This was an ambitious study and I applaud the authors for the work that was done to address not just my own concerns but also the concerns of the other reviewers. I believe that the paper is much stronger and has a clear and theoretically supported narrative.
With these changes I am satisfied. I have no further requests.

Response 1.0: We thank you for your time and encouraging evaluation of our manuscript.

Reviewer #2 (Remarks to the Author):

The authors have put considerable effort into this revision, particularly by adding important analyses (e.g., a leave-one-site-out cross-validation across 5 studies, plus additional generalization tests) and clarifying crucial methodological choices. This work has resulted in an overall improved manuscript.

At the same time, despite these new results, I remain skeptical about the depth of the authors' interpretation of the findings. As noted in my initial evaluation, my first concern is the interpretation of the results as a selective neural signature of mentalizing. As the authors themselves report in the revised manuscript, their findings provide initial evidence of specificity by showing that the signatures preferentially predict inferences about "hidden" internal states relative to factual judgments, particularly in social contexts. However, above-chance performance also appears in nonsocial attributional conditions. This point should be discussed in the manuscript, and not only in the Response letter. Given the explicit aim of the study and the emphasis on "BRAIN SIGNATURES OF MENTALIZING," in my view this consideration should be expressed in a brief Limitations section explicitly delimiting claims of specificity. In addition, references to "specificity/selectivity" should be softened across the abstract, results, and discussion.

A similar concern holds for the acknowledgment that the observed "neural signature" pertains primarily to trait-based mentalizing (i.e., mentalizing about personality traits). While this is now acknowledged in the Introduction and Discussion, I think it should be made explicit in the Abstract with just one more word (if not the title) to more accurately reflect the scope of the authors' inference.

Response 2.0: We thank you for your time, constructive comments, and encouraging evaluation of our manuscript. To address your remaining concerns, we have now made several additional changes.

First, to address the concern regarding the selectivity/specificity of the mentalizing signatures, we have now added additional classification analyses for social vs. nonsocial conditions and further edited the manuscript to more thoroughly discuss the performance in nonsocial conditions.

Averaging across attributional and factual conditions, we tested how well the Mentalizing Signature (MS) and the Other-Referential Signature (Other-RS) predict social against nonsocial statements. The MS clearly separated social from non-social inferences, whereas the Other-RS did not (see panel B below). We have updated **Fig. 6** to incorporate this additional analysis, as shown below.

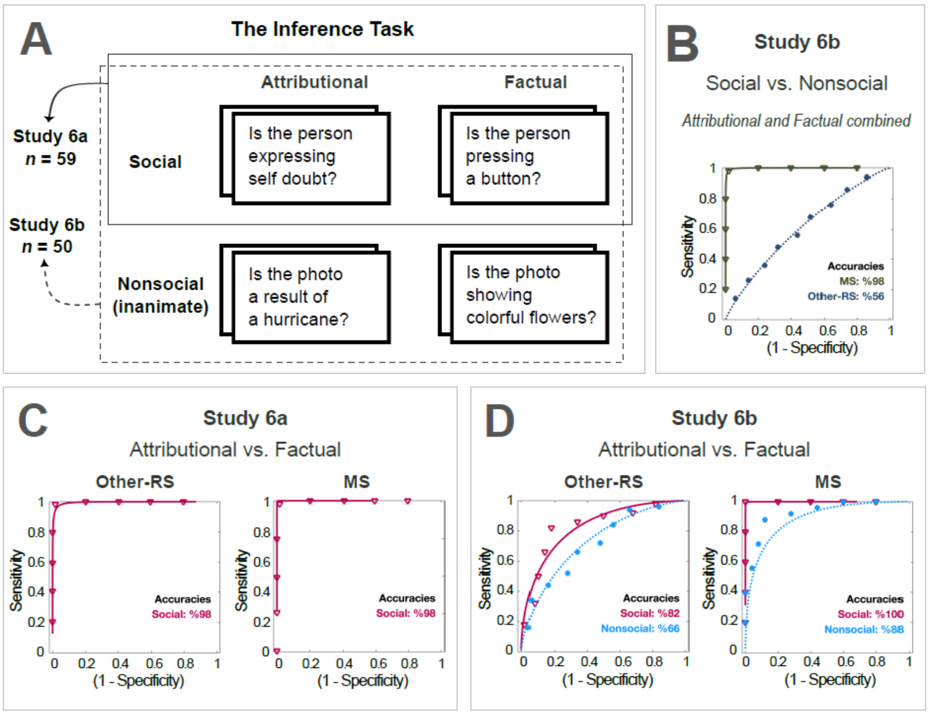

In addition, we tested the predictions of the LOSiO (Leave-one-site-one) classifiers in the inference task and found them to be the most sensitive to attributional statements in the social context. However, the MS_{LOSiO} also captured nonsocial attributional processing to some extent. We added these analyses to the revised manuscript (see Fig. 9E below):

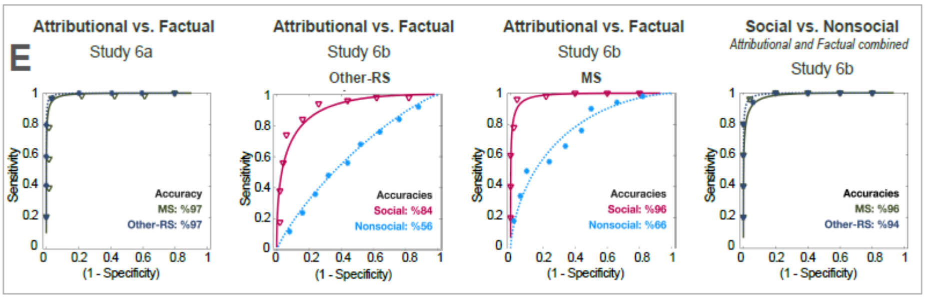

- **See also in Results, line 328:** *"Further, we tested how well the signatures separate social from nonsocial inferences (averaged across attributional and factual conditions). While the Other-RS did not significantly separate social from non-social inferences (56% accuracy, p = .48, Cohen's d = 0.27, AUC = 0.62, sensitivity = 56%, specificity = 56%), the MS separated social from nonsocial inferences with almost perfect accuracy*

(98% correct, p < .001, Cohen's d = 2.96, AUC = 1.00, sensitivity = 98%, specificity = 98%; see Fig. 6B)."

- **And line 433: "***Finally, we applied the predictions of the LOSiO classifiers to the inference task (Study 6) to test how well they classify attributional versus factual and social versus nonsocial conditions (see Fig. 9E). Both Other-RS_{LOSiO} and MS_{LOSiO} predicted social attributional (versus factual) statements in Study 6A with 97% accuracy (p < .0001; Cohen's d for Other-RS_{LOSiO} = 2.75 and for MS_{LOSiO} = 2.42). In Study 6b, the Other-RS_{LOSiO} separated attributional versus factual statements in the social condition with 84% accuracy (p <.001, Cohen's d = 1.32), whereas this classification was non-significant in the nonsocial condition (accuracy = 56%, p = .48). The MS_{LOSiO} predicted attributional versus factual statements in the social condition with 96% accuracy p < .001, Cohen's d = 2.16) and in the nonsocial condition with 66% accuracy (p = .033, Cohen's d = 0.73). Finally, MS_{LOSiO} and Other-RS_{LOSiO} separated social from non-social inferences with 96% and 94% accuracy, respectively (p <.001, Cohen's d for MS_{LOSiO} = 2.12, and for Other-RS_{LOSiO} = 2.5). Together, this illustrates that, similar to the mentalizing signatures, the LOSiO classifiers are most sensitive to attributional processing in the social context, while also capturing nonsocial attributional processing to some degree."*
- **Text edit in Discussion, line 475**: *"It [the MS] performed very well in separating higher-level inferences about others and hidden states from factual inferences, with higher classification in social compared to non-social contexts. The MS (but not the Other-RS) also significantly separated social from nonsocial inferences. Together, these findings demonstrate its sensitivity to mental state compared to external state attributions."*
- **A new paragraph in Discussion, line 546**: *"Of note, the mentalizing signatures were also implicated in making inferences about nonsocial targets – albeit to a lesser degree compared to social targets (see Fig. 6D and Fig 9E). An earlier study (Spunt & Adolphs, 2015) that used a similar inference task as Study 6 found that social and nonsocial attributions displayed overlapping activations in dmPFC, STS, TPJ, and lateral orbitofrontal cortex. A common denominator in both conditions may reflect domain-general attributional processing (Spunt & Adolphs, 2017), which at least partially builds on semantic cognition (Binney & Ramsey, 2020). Likewise, processes supporting mentalizing may also more broadly subserve extraction of conceptual and semantic meaning for nonsocial stimuli (Andrews-Hanna & Grilli, 2021; Baetens et al., 2014), in line with the default mode network's putative meaning-making function (Andrews-Hanna et al., 2014; Yeshurun et al., 2021, Koban, et al., 2021). Thus, the mentalizing signatures may involve components of both domain-general representational processing, domain-specific social processing, and target-specific (self and other) processing components."*
- **Highlighting this issue in the limitations section (line 588)**: *"However, they also show above-chance classification in nonsocial inferences (see Fig 6D and Fig. 9E), which might raise concerns about their specificity to social agents. Future studies are needed to further test how well the mentalizing signatures separate mentalizing about other agents from other higher-order, conceptual, and abstract cognitive processing. Future studies should also test their specificity, as well as their validity in other forms (e.g., affective, spontaneous, or non-verbal) of mentalizing."*

Second, we agree with your recommendation to better clarify the scope of our paper in the Abstract. Following this advice, we edited the manuscript to highlight the trait-based aspect of mentalizing as follows (edits underlined):

- **Abstract (line 64)**: *"Together, our findings reveal consistent and* separable neural patterns subserving trait-based mentalizing about self and others…"
- **Introduction (line 164): "***We first used standard machine learning algorithms—support vector machines—to develop and cross-validate multivariate classifiers (signatures) of mentalizing about traits in a sample of healthy adults."*
- **Discussion, line 455**: *"The four signatures showed good to excellent classification accuracies in independent data and provided several insights into the brain circuits of trait-based mentalizing."*

Reviewer #3 (Remarks to the Author):

I appreciate the authors' detailed and thorough replies to the comments and the additional analyses they have performed. Many of the secondary issues I raised have been addressed.

Response 3.0: We thank you for your time and acknowledgement that many of your concerns have been addressed by our revision.

However, my principal concern remains unresolved and, in my view, continues to be a critical flaw in the methodological foundation of the work. Specifically, the authors use subject-level GLM contrast maps as the sole input to the SVM classifiers. While they justify this as standard practice, I remain unconvinced that this approach is appropriate for the claims made in this paper. While I agree with the authors that preprocessing fMRI data is absolutely necessary, and their rationale for steps such as motion correction, normalization, and filtering is sound, the inclusion of a full first-level GLM analysis within what they describe as preprocessing is problematic. GLM modeling is not a preprocessing step; it is already a statistical analysis that imposes strong assumptions and extracts contrasts of interest. By feeding GLM-derived contrast maps into the SVM, the analysis applies machine learning to data that have already been statistically filtered for their relationship to the experimental conditions. While this may not constitute circularity in the classical sense, it raises concerns about analytical redundancy and makes it difficult to determine what unique information the SVM classification provides beyond the GLM analysis itself.

Response 3.1: To the best of our understanding, you are not convinced that including a first-level analysis step before training SVMs is necessary and therefore concerned about analytical redundancy. You specifically raise concerns about i) the use of first-level analysis before SVM and ii) the added value of SVM following a GLM analysis. We respond to the latter in Response 3.3.

Regarding your first point, as we explained briefly in the manuscript, using GLM-derived beta maps as classifier inputs is the standard practice in fMRI decoding (Haynes, 2015, Neuron; Scheinost et al., 2019, NeuroImage) and the brain signatures approach employed here and in several influential previous papers (Wager et al., 2013, NEJM; Woo et al., 2014, Nat Comms; Kragel et al., 2018, Neuron; López-Solà et al., 2017, Pain; Koban et al., 2023, Nat Neurosci). The essential rationale behind this is that a first-level GLM analysis is a necessary step to remove the structured noise and isolate the signal of interest, which, in

this case, are the condition-specific, voxel-wise activity estimates. On the other hand, any model that does not handle temporal autocorrelation, trial structure, and nuisance effects would be uninterpretable and dominated by noise rather than by experimental effects. To be clear, first-level GLM and group-level classifier training are independent steps of the analysis. First-level GLM contrasts conditions on the individual level producing voxel-wise estimates of task conditions. Group-level classifier training (SVM) develops predictive weight maps that best work in the training dataset, which we then further test in the independent datasets. Our goal is to obtain classifiers that are valid at the population-level, rather than at an individual- or group-level. First-level GLM (on the individual participant level) and training SVMs (on the group level) therefore represent two fully independent and non-circular steps.

We were somewhat bewildered by the suggestion that we portrayed first-level GLM analyses as pre-processing. We obviously concur that pre-processing and first-level GLM analyses are unquestionably different steps, and we describe them in the paper, accordingly:

- Line 747: *"SPM8 (Wellcome Department of Imaging Neuroscience, UCL, London, UK) and Matlab® (The MathWorks Inc.) were used for image preprocessing and first-level analysis."*
- Line 769*: "Please refer to the original studies (see Supplementary Table 1) for details of image acquisition, preprocessing, and first-level analysis."*

However, we acknowledge that this false impression might have arisen from the ordering of titles in the Methods section. Thus, to accommodate for this potential for misunderstanding, we updated the relevant title as follows: *"fMRI data acquisition, preprocessing, and first-level analysis"* (line 735).

Moreover, adding a layer of machine learning analysis does not address the underlying limitations of the GLM framework. The intrinsic correlational nature of the analysis remains, and causal inferences about neural mechanisms cannot be drawn from either approach.

Response 3.2: We understand this concern, however it does not apply to our manuscript, to the best of our knowledge. We would like to emphasize that the main goal of this paper is not to infer causality in terms of region-condition associations, but to develop generalizable, predictive neural signatures that can be applied easily to new datasets and studies. We achieved this goal sufficiently, as demonstrated by the very good classification performances across the independent testing datasets described in our paper. Moreover, we have paid careful attention to refraining from any implications of causality throughout the paper.

Furthermore, in practice, this reduces the classification step to a group-level analysis of GLM-derived maps. Thus, it remains unclear to me what this method provides that could not be achieved through a conventional second-level GLM. If the results from explaining the SVM are comparable to a second-level GLM (the voxels on the first-level maps that are more common for each condition across subjects), I continue to ask myself the question "what is the scientific advancement brought about by this method?", but I still could not find a reassuring answer. The manuscript would be strengthened if the authors could more clearly articulate the unique insights this SVM approach provides beyond conventional second-level GLM analyses. A direct comparison with second-level GLM results would help demonstrate the added value.

Response 3.3: We agree with you that it is important to clarify the added value of SVM. Our primary goal in this study was to develop multivariate brain models (see Kragel, Koban, et al., 2018, Neuron) of self and other-related mentalizing that are replicable and generalizable. This requires a machine learning solution approach over a standard GLM.

Specifically, it should be highlighted that, while encoding models (e.g., standard second-level GLM) aim at explaining brain activity from known features of a stimulus, decoding approaches (e.g., multivariate classifiers) aim at inferring stimulus/mental state/condition from brain responses (see Kragel, Koban, et al. 2018, Neuron for a review). Therefore, the advancement brought by the use of a decoding approach in our paper is to be able to predict conditions given the brain responses, which for instance, showed promise for clinical use, as illustrated by the lower self/other condition differentiations in schizophrenia patients versus healthy adults in our paper.

In order to clearly articulate the added value of our approach, we included an additional statement in Introduction (see line 101) within two paragraphs where we address this very question (edits underlined):

> (Line 91): *"Many studies have examined the neural correlates of mentalizing, suggesting an interplay of different brain regions, including medial prefrontal cortex (mPFC), temporoparietal junction (TPJ), and precuneus[10–14]. However, predictive brain measures of mentalizing that can be easily applied to new data to decode the degree of self-related and other-related processing are still lacking. Most cognitive and affective processes cannot be captured by activity in individual brain regions, as they are reflected in patterns of brain activity distributed across multiple regions and systems, which can be harnessed by pattern-based decoding[15,16]. For instance, recent work demonstrates that distributed brain activity and connectivity patterns, as indexed by fMRI, enable us to decode the intensity of pain[17], drug and food craving[18], sustained attention[19], depressive rumination[20], and clinically relevant behaviors and outcomes[21,22]. As opposed to the standard brain mapping approach that maps task features onto brain activity, these predictive brain activity patterns or 'brain signatures' are multivariate models that utilize data across the whole brain to make formally testable population-level predictions across subjects and datasets[15,23]. The predictions address the involvement and/or the intensity of a mental process. Here, we apply this brain signature approach to predict mentalizing about oneself or other people.*
>
> *Mentalizing, as a hidden state, would arguably benefit from such an approach. Thinking about self and others is inherently multilayered and multidimensional[12,24,25]. Recent conceptualizations of self- and social-cognition point to multiple dimensions, such as about self versus others, affective versus cognitive[6,12,26] and multiple layers, such as observing behaviours, and processing at the state and trait levels[24]. Moreover, social cognition terms, including but not limited to mentalizing (e.g., empathy, perspective taking), suffer from inconsistent usage in the literature and a lack of consensual definitions[27]. Developing brain signatures of mentalizing could potentially help account for this heterogeneity, offering the possibility of testing whether the proposed subdimensions of mentalizing are subserved by dissociable neurobiological patterns. In turn, these signatures carry the potential for validating the multiple facets of mentalizing[15]. However, it remains unclear—especially considering the complexity of the mentalizing construct—whether distributed neural patterns can reliably predict mentalizing using brain images."*

Lastly, regarding the usage of masks in the analysis. I still do not see a valid reason for using

the “very comprehensive” social cognition mask. In my opinion, the use of a theory-driven mask raises concerns about the validity of any relevant conclusions regarding the importance of each voxel; i.e., circularity persists in the method as voxels are previously screened prior to the application of the statistical tests. Some conclusions may be taken, such as whether a voxel contributes positively or negatively. However, as the authors note, the use of the mask restricts voxels to known ToM areas, thereby precluding the model from capturing potentially relevant activations in less traditional regions, besides inflating the probability of those pertaining to the masks. Therefore, in my opinion, the study now added to the supplementary materials section is much more relevant than the main study of this article, since it uses a whole-brain gray matter mask (the usage of a gray matter mask is biologically plausible, as there should not be BOLD signal in white matter).

Response 3.4: We understand this concern, which we have fully addressed in our previous revision. We have presented clear evidence in the previous revision that alternative strategies, such as whole-brain mask or recursive feature elimination (RFE), lead to very similar predictive performances and brain weights. The main scope of this paper was self- vs other-related mentalizing, both of which are parts of the overall mentalizing network. Thus, there should not be any bias in the inclusion of regions in either of these conditions. On the contrary, if there had been a bias in the identification of regions in mentalizing vs non-mentalizing conditions, this would have been reflected in our classification rates in independent samples, which are exceptionally high especially for the overall mentalizing signature. Therefore, this represents an analytic choice (that we think will allow for better generalizability in future studies) rather than an analytic error.

For these reasons, despite the otherwise comprehensive revisions, I continue to raise serious concerns about the core of the manuscript maintaining, therefore, the same recommendation.

José Paulo Marques dos Santos
